# The in vitro gastrointestinal digestion-associated protein corona of polystyrene nano- and microplastics increases their uptake by human THP-1-derived macrophages

**DOI:** 10.1186/s12989-024-00563-z

**Published:** 2024-02-04

**Authors:** Hugo Brouwer, Mojtaba Porbahaie, Sjef Boeren, Mathias Busch, Hans Bouwmeester

**Affiliations:** 1 Division of Toxicology, Wageningen University, Stippeneng 4, 6708 WE Wageningen, The Netherlands; 2 Laboratory of Biochemistry, Wageningen University, Wageningen, The Netherlands; 3 Laboratory of Cell Biology and Immunology, Wageningen University, Wageningen, The Netherlands

**Keywords:** Macrophage, In vitro gastrointestinal digestion, Polystyrene, THP-1, Endocytosis, Protein corona, LC‒MS–MS, Proteomics

## Abstract

**Background:**

Micro- and nanoplastics (MNPs) represent one of the most widespread environmental pollutants of the twenty-first century to which all humans are orally exposed. Upon ingestion, MNPs pass harsh biochemical conditions within the gastrointestinal tract, causing a unique protein corona on the MNP surface. Little is known about the digestion-associated protein corona and its impact on the cellular uptake of MNPs. Here, we systematically studied the influence of gastrointestinal digestion on the cellular uptake of neutral and charged polystyrene MNPs using THP-1-derived macrophages.

**Results:**

The protein corona composition was quantified using LC‒MS–MS-based proteomics, and the cellular uptake of MNPs was determined using flow cytometry and confocal microscopy. Gastrointestinal digestion resulted in a distinct protein corona on MNPs that was retained in serum-containing cell culture medium. Digestion increased the uptake of uncharged MNPs below 500 nm by 4.0–6.1-fold but did not affect the uptake of larger sized or charged MNPs. Forty proteins showed a good correlation between protein abundance and MNP uptake, including coagulation factors, apolipoproteins and vitronectin.

**Conclusion:**

This study provides quantitative data on the presence of gastrointestinal proteins on MNPs and relates this to cellular uptake, underpinning the need to include the protein corona in hazard assessment of MNPs.

**Graphical abstract:**

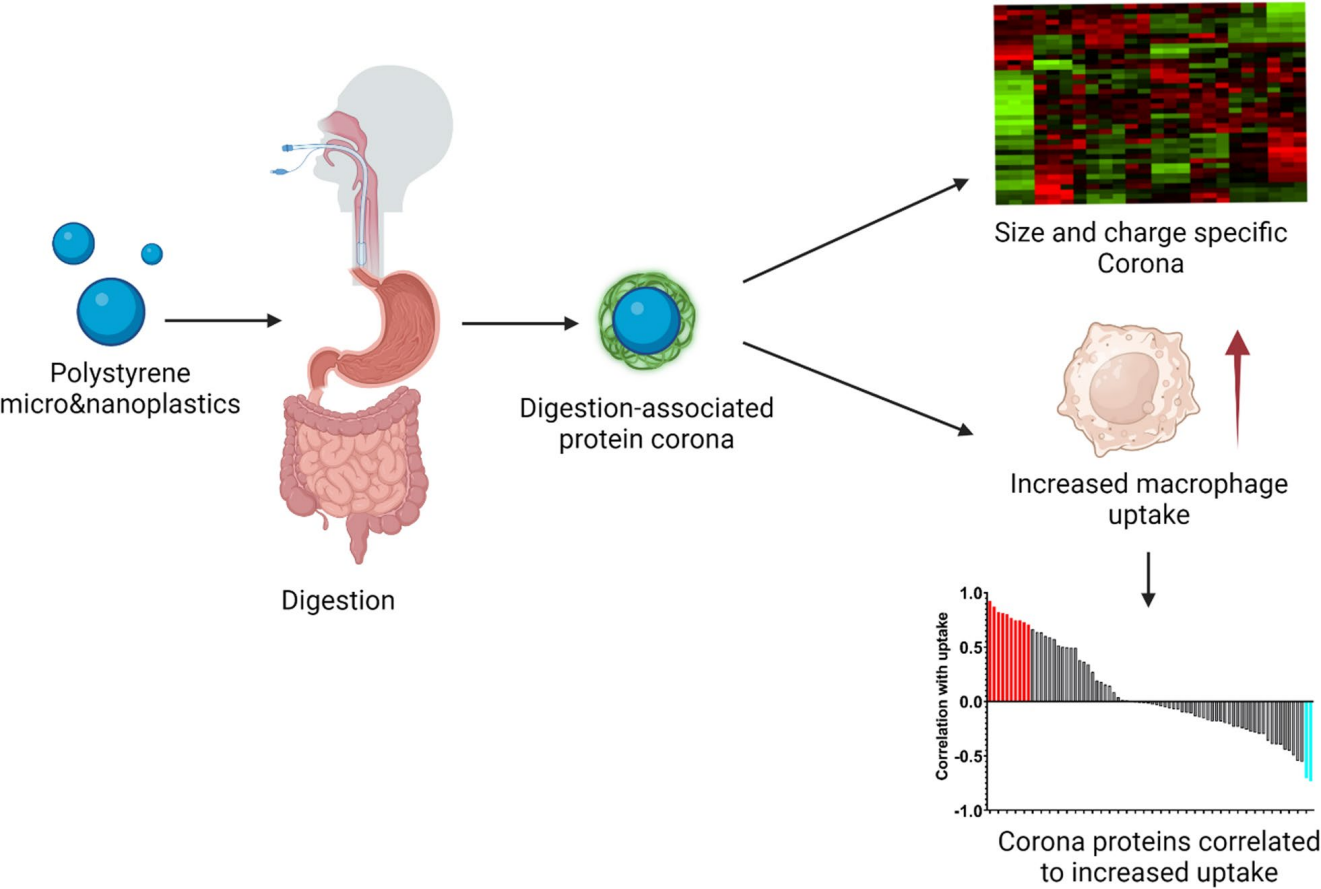

**Supplementary Information:**

The online version contains supplementary material available at 10.1186/s12989-024-00563-z.

## Highlights

- In vitro digestion increases uptake of nanoplastics by macrophages.
- The effect on uptake is dependent on particle size and charge.
- Uptake differences could be related to alterations in the protein corona.
- Uptake differences were strongly correlated to digestion associated alterations in complement factor and apolipoprotein levels in the protein corona.

**Supplementary Information:**

The online version contains supplementary material available at 10.1186/s12989-024-00563-z.

## Background

Micro- and nanoplastics (MNPs) are one of the most widespread pollutants in the environment and are considered one of the five most concerning contaminants in food [1]. MNPs are mostly generated from plastic waste spilled into the environment where its continuous exposure to chemical and physical stress, such as abrasion or UV light, causes the plastic waste to fragment into smaller particles [1]. A second source of environmental MNPs arises from MNPs that are intentionally added to industrial and consumer products and spill into the environment during the product life cycle [2, 3]. Plastic particles with a size < 5 mm are defined as microplastics, while particles with a size < 100 nm are defined as nanoplastics [1, 4]. Currently, MNPs have been detected in virtually all environmental compartments [5], including inside and outside air [6], in household dust [7], and in food commodities, including meat [1, 5], honey [8], salt [9], shellfish [10], fish [11], beer [12] and drinking water [13]. Human exposure to MNPs by ingestion or inhalation is thus unavoidable, and reports indicating the presence of MNPs in human blood and placenta highlight the ubiquity of human exposure and the systemic availability of MNPs [14, 15].

MNP exposure via ingestion (including swallowing of respirable MNPs trapped in mucus cleared from the lungs) is the dominant route of exposure for humans [16]. Before MNPs reach the intestinal epithelial barrier, they pass through the stomach and intestinal luminal content, which is dynamic in pH and ionic strength and contains complex digestive fluids and food-derived molecules. This exposure results in spontaneous coverage of the MNP surface by a complex cocktail of intestinal proteins and peptides, which is collectively referred to as the protein corona [17, 18]. The protein corona has been shown to affect the interaction of MNPs with cells either by shielding the reactive surface of the MNPs or by facilitating uptake through membrane receptor interactions [19–21]. Importantly, the presence of a protein corona and its composition have been shown to be more predictive of cellular effects than nanoparticle properties themselves [18, 22]. Despite recognition of the importance of the protein corona for the hazards of engineered (metal) nanomaterials [18, 23–25], little is known about the influence of gastrointestinal digestion on the protein corona on MNPs or on the resulting biological impact. Previously, we showed that the cellular association of 50 nm polystyrene (PS) MNPs [23, 26] and 50 nm silver nanoparticles [23] with Caco-2 cells was increased after in vitro gastrointestinal digestion. However, the implications for larger-sized MNPs are unknown. Given the great diversity of MNPs that consumers can be exposed to, we studied the relationship between in vitro gastrointestinal digestion of well-characterized PS MNPs of different sizes and surface charge and their cellular uptake. By doing so, we address the need for systematic data required to implement read-across methodologies based on the particle size and surface properties and relate this to the cellular uptake needed to advance MNP hazard assessment [27–30].

A realistic intestinal protein corona can be created on MNPs using well-established in vitro gastrointestinal digestion approaches. In this study, we used the validated and standardized INFOGEST-2 protocol as the basis for the in vitro digestion protocol [31]. Several cell-based in vitro models can be used to assess the impact of the protein corona on cellular uptake. In vivo, the intestinal uptake of most MNPs is thought to be facilitated by M-cells, which have a high phagocytotic capacity [32]. Yet current cell culture protocols cannot produce M-cells in the absence of other cell types, and no protocols are available that generate M-cells that show surface marker expression similar to that of in vivo M-cells [30, 33]. For this reason, we decided to use a THP-1-derived macrophage model emulating tissue-resident macrophages that are located downstream of M-cells and thus represent a highly exposed cell population. Furthermore, macrophages are capable of accumulating nanoplastics [34, 35] and are the main cell type involved in systemic transport and blood clearance of MNPs [36–44]. Therefore, uptake kinetics obtained using THP-1-derived macrophages can be used to estimate endocytosis uptake by cells present in highly exposed tissues in vivo.

The aim of the current study was to assess the impact of in vitro gastrointestinal digestion on the MNP protein corona composition and its consequences on uptake by THP-1-derived macrophages. To study this, we used seven representative PS MNPs with different sizes and surface charges. PS MNPs were digested in vitro using a modified INFOGEST-2 protocol 31, and the resulting protein corona was analyzed using SDS‒PAGE and LC‒MS–MS. THP-1 derived macrophages were subsequently exposed to noncytotoxic PS MNP concentrations, and flow cytometry combined with confocal microscopy was used to determine PS MNP uptake. Finally, the protein abundance in the corona was correlated with particle uptake to investigate which proteins play a role in PS MNP internalization.

## Methods

### Materials

Phosphate-buffered saline (PBS), paraformaldehyde, potassium chloride, sodium bicarbonate, sodium chloride, monopotassium phosphate, magnesium dichloride hexahydrate, ammonium carbonate, calcium chloride dihydrate, hydrochloric acid, bovine bile, porcine pepsin, porcine pancreatin and p-toluene-sulfonyl-L-arginine methyl ester (TAME) were obtained from Merck (Amsterdam, Netherlands). Laemmli sample buffer (2 ×) was obtained from Bio-Rad (Lunteren, The Netherlands). Non-heat inactivated non-irradiated fetal bovine calf serum(FBS) with product number FBS-12a was obtained from Capricorn Scientific (Ebsdorfergrund, Germany). Trifluoroacetic acid, dithiothreitol and iodoacetamide were obtained from Sigma Aldrich (Zwijndrecht, The Netherlands), and sequencing grade trypsin was obtained from Boehringer Mannheim (Mannheim, Germany). Precast SurePAGE MOPS-Tris PAGE gels were obtained from Genscript (Rijswijk, The Netherlands). Alexa-fluor 594-conjugated wheat germ agglutinin was obtained from Thermo Fisher (Bremen, Germany).

### Particles

Green fluorescent (Ex 441, Em 485) and nonfluorescent polystyrene (PS) MNPs (50 nm, 100 nm, 200 nm, 500 nm, 1000 nm) were obtained from Polysciences (Hirschberg an der Bergstraße, Germany). Green fluorescent and nonfluorescent carboxyl- and amine-modified PS MNPs (100 nm) were obtained from Magsphere (Pasadena, USA). Throughout the rest of the manuscript, the particles will be referred to as shown in Table 1.

**Table 1:** MNPs used in this study with the respective abbreviations

Particle	Abbreviations
Polystyrene, 50 nm	PS50
Polystyrene, 100 nm	PS100
Amine-modified polystyrene, positive charge, 100 nm	PS100 (+)
Carboxyl-modified polystyrene, negative charge, 100 nm	PS100 (−)
Polystyrene, 200 nm	PS200
Polystyrene, 500 nm	PS500
Polystyrene, 1000 nm	PS1000

### In vitro* gastrointestinal digestion of MNPs*

For the in vitro gastrointestinal digestion, the INFOGEST-2 protocol [31] was used with minor modifications. Briefly, the digestion protocol consists of 3 steps, namely, the sequential addition of simulated salivary fluid (SSF), simulated gastric fluid (SGF) and simulated intestinal fluid (SIF) to the sample of interest, representing the oral, gastric and intestinal digestive phases, respectively (Table 2).

**Table 2:** Composition of digestive fluids

Component	Simulated salivary fluid (SSF) (pH = 7.0)	Simulated gastric fluid (SGF) (pH = 3.0)	Simulated intestinal fluid (SIF) (pH = 7.0)
KCl	30.2 mM	6.9 mM	6.8 mM
NaHCO_3_	27.2 mM	25.0 mM	85.0 mM
NaCl	–	47.2 mM	38.4 mM
KH_2_PO_4_	7.4 mM	0.9 mM	0.8 mM
MgCl_2_. (H_2_O)_6_	0.3 mM	0.1 mM	0.3 mM
(NH_4_)_2_CO_3_	0.1 mM	0.5 mM	–
CaCl_2_(H_2_O)_2_	1.5 mM	0.15 mM	0.6 mM
Pepsin	–	0.8 mg/ml	–
Bovine Bile	–	–	4.0 mg/ml
Porcine Pancreatin	–	–	14.0 mg/ml

After dissolution of all the salts, the pH of the SSF, SGF and SIF was adjusted to 7.0 ± 0.5, 3.0 ± 0.5, and 7.0 ± 0.5 for each fluid, respectively, using 1 mM HCl, and subsequently, all fluids were autoclaved. Prior to in vitro gastrointestinal digestion, 1000 U/ml pepsin was added to SGF, while 4 mg/ml bile and 100 U/ml pancreatin were added to SIF. Bile and pancreatin were dissolved by incubation at room temperature (RT) while rotating head-over-heels for 1 h (at 25 RPM), and pepsin was dissolved by head-over-heels rotation for 5 min at RT. After addition of the proteins, the SIF was centrifuged at 3000 g for 5 min to remove undissolved pancreatin fibers and bile. No loss in enzyme activity was observed upon centrifugation using a trypsin activity assay as described in the INFOGEST2 protocol [31] (data not shown). Just prior to the in vitro gastrointestinal digestion, a 0.3 M CaCl_2_ solution was added to simulated salivary, gastric and intestinal fluid in 1:200th, 1:2000th and 1:500th part respectively.

At the start of the digestion, 125 µl of a 2.5% MNP suspension (i.e., 3.125 mg total MNP mass) was sonicated in an ultrasonic bath (VWR, Amsterdam, Netherlands) to ensure a homogeneous suspension and was added to a 2 mL Eppendorf vial. First, 125 µl of 2X concentrated SSF was added to the MNPs in each sample, followed by manual mixing through inversion and a 5 min incubation at RT. Then, 250 µl of SGF was added to each sample, and the pH was adjusted to pH 3.0 ± 0.5 using 1 mM HCl. The samples were incubated at 37 °C for 2 h while rotating head-over-heels at 25 RPM. Finally, 500 µl of SIF was added to each sample and the pH was adjusted to 7.0 ± 0.5 using 1 mM HCl, and the samples were incubated for 2 h at 37 °C while rotating head-over-heels. For each experiment, a digestion blank including demi-water instead of MNP suspension was included. The digestion matrix needed to be diluted 25X in complete cell culture medium (CCM) before cell exposure to prevent matrix cytotoxicity (Additional file 1: Fig. S1).

To assess the protein corona formed upon in vitro gastrointestinal digestion or upon incubation (of nondigested) PS MNPs in CCM, PS MNPs were also incubated in CCM for 2 h while rotating head-over-heels at 37 °C. We will refer to PS MNPs that were suspended in serum-free medium as pristine MNPs and those that were subjected to in vitro gastrointestinal digestion as digested MNPs. PS MNPs that were incubated in FCS-containing CCM will be referred to as serum-coated MNPs, and PS MNPs that underwent digestion and subsequent incubation in CCM will be referred to as digested+serum-coated MNPs (see Table 3). After digestion or incubation with serum, the samples were used immediately for MNP characterization or cell exposure.

**Table﻿ 3:** Nomenclature of MNPs according to their pretreatment and incubation in medium

	Without in vitro intestinal digestion	Upon in vitro intestinal digestion
Medium without serum	Pristine MNPs	Digested MNPs
Medium with serum	Serum-coated MNPs	Digested+serum-coated MNPs

### Characterization of particle suspensions

The hydrodynamic sizes of the PS MNPs before and after in vitro gastrointestinal digestion were determined by dynamic light scattering (DLS) using a ZS-nano zetasizer (Malvern Panalytical, Malvern, Great Britain). Cell culture medium without serum was filtered through a 0.2 µm nylon Whatman filter to remove background particulate matter. Pristine MNPs, digested+serum-coated MNPs and serum-coated MNPs were diluted to a concentration of 125 µg/ml in serum-free cell culture medium. The suspensions were sonicated for 1 min using a sonication bath and were subsequently loaded into a 1.5 mL polystyrene cuvette. The particle sizes were determined under a scattering angle of 173° and at a temperature of 25 °C. At least 5 autocorrelation curves were obtained for each sample, and all particles were measured in triplicate. The remaining zeta-sizer settings were left at their default value. For each set of measurements, a medium blank was included to determine background particulate matter. Data were analyzed using two-way ANOVA with Bonferroni post-hoc correction using graphpad prism 9.

To determine potential fluorophore leaching from the MNPs upon in vitro gastrointestinal digestion, MNPs were removed from the gastrointestinal digestion supernatant using centrifugation at 30,000 g for 30 min, and the fluorescence intensity of the supernatant was measured using a Spectramax iD3 Multi-Mode Microplate Reader (Molecular Devices, Birkshire, United Kingdom). Leaching of fluorophores from the MNPs was not detected (Additional file 1: Fig. S2).

### Assessing particle dosimetry

We assessed the fraction of MNPs deposited on the cells using the in vitro sedimentation, diffusion and dosimetry (ISDD) model [45]. The ISDD model was run assuming a particle density of 1.05 g/ml, and assuming no agglomerate formation, the effect of protein binding was not considered for predicting particle sedimentation. The column height was set to 2.631 mm (corresponding to a 48-well plate format), and the medium volume was set to 0.5 ml. The grid was set to consist of 300 compartments, and sedimentation up to 24 h after cell exposure was simulated. The remaining settings were left at their default conditions. The predicted deposited dose is shown in the Supplementary materials (Additional file 1: Fig. S3).

### Cell culture

THP-1 cells were obtained from ATCC (Manassas, USA) and were grown in RPMI 1640 medium (A10491, Thermo Fisher, Waltham, USA) supplemented with 10% fetal calf serum and 1% penicillin/streptomycin. The cells were cultured in a humidified incubator at 37 °C at 5% CO_2_. Cells were maintained at a cell density between 2*10^5^ and 8*10^5^ cells/ml in an upright T75 grainer culture flask and were passaged twice a week. THP-1 cells were seeded at 5*10^5^ cells/ml in all experiments. For cell viability experiments, 100 µl THP-1 cells were seeded in 96-well plates and subsequently differentiated to M0 macrophages by the addition of 20 ng/ml phorbol 12-myristate 13-acetate (PMA) and were incubated for 48 h in a humidified incubator [46]. After that, the PMA-containing medium was replaced with PMA-free complete cell culture medium, and the cells were allowed to rest for 24 h. On the subsequent day, the cells were exposed to MNPs or blanks for up to 24 h.

### Cell viability assessment

To derive noncytotoxic concentrations of the gastrointestinal digestion matrix and MNPs to THP-1-derived macrophages, a water-soluble tetrazolium dye 1 (WST-1) assay was performed. Briefly, THP-1 cells were seeded and differentiated to macrophages in 96-well plates as described above (see Sect. "Cell culture"). Only nonfluorescent MNPs were used for the cell viability assay to prevent potential interference of the fluorescent label with the WST-1 viability assay. Based on the cytotoxicity of the digestion matrix (Additional file 1: Fig. S1), the digested MNPs and serum-coated MNPs were diluted 1:25 with complete culture medium to a concentration of 125 µg/ml. THP-1-derived macrophages were exposed to digested+serum-coated-MNPs or serum-coated-MNPs at concentrations of 7.8, 15.6, 31.3, 62.5, and 125 µg/ml for 24 h. Next, the medium was aspirated, and the cells were washed with PBS to remove MNPs that were not associated with the cells. Then, 100 µl of culture medium containing 5% WST-1 reagent was added to each well, and the plates were incubated for 1 h at 37 °C while shaking at 300 RPM. After 1 h, the absorbance at 440 nm was measured using a Spectramax iD3 Multi-Mode Microplate Reader. The MNPs showed no signal in a cell-free WST-1 assay (data not shown), excluding potential particle interference.

For each experiment, a positive control containing 0.5% Triton X-100 and a negative control consisting of complete culture medium were included. The viability was calculated by comparing the absorbance at 440 nm to the negative control and expressing the resulting absorbance as a percentage of the negative control. The data were analyzed using GraphPad Prism 9.3.1 (GraphPad Software, LLC, San Diego, CA, USA), and a two-way ANOVA using Bonferroni post hoc correction was performed to assess significant differences from the medium control. Experiments were performed in triplicate.

### Quantitative measurements of cell association

MNP cell association was measured using flow cytometry. Briefly, THP-1 cells were seeded in a 48-well Nunclon plate (Thermo Fisher) and were differentiated to macrophages as described above (see Sect. "Cell culture"). The THP-1-derived macrophages were exposed to digested+serum-coated or serum-coated MNPs for up to 24 h. The cells were subsequently fixed using 4% paraformaldehyde in PBS for 15 min at room temperature and washed twice using PBS. After fixation, the cells were stored at 4 °C until measurement using flow cytometry for at most 24 h. Prior to flow cytometry, 1 mM EDTA was added to the cell suspension, and the cells were detached from the bottom of the well using a curved Pasteur pipette and transferred to a 96-well Nunclon plate. Cell fluorescence was measured in triplicate, each consisting of 3 technical replicates, using a Cytoflex LX flow cytometer in plate mode (Beckman Coulter NL, Woerden, Netherlands). MNP cell association was assessed based on the fluorescein isothiocyanate (FITC) channel (525 nm:20), which corresponds to the green fluorescence emitted by the MNPs. Cells were distinguished from cell debris based on their forward and side scatter, singlet cells were distinguished based on their forward scatter height and width and at least 5000 cells were analyzed for each of the samples. Cells that had a FITC signal higher than 99% of the cells in the negative control were considered FITC positive. The cell association of MNPs over time of two different concentrations of MNPs (i.e., 15.6 and 62.5 µg/ml) was analyzed to determine the optimal measurement time (Additional file 1: Fig. S4 & S5). Next, the cell association upon 24 h incubation with 7.8, 15.6, 31.3, 62.5 or 125 µg MNPs/ml was assessed (n = 3). To correct for differences in fluorescence intensity between the different MNPs, the obtained relative light unit (RLU) values were divided by the relative fluorescence intensity of each of the MNPs at 525 nm as determined using a luminometer (Additional file 1: Fig. S6). Then, the corrected fluorescence was expressed as the fold change compared to the average fluorescence intensity of unexposed cells. The flow cytometry sorting panel is shown in Additional file 1: Figure S8.

### Determination of cellular internalization

Internalization of fluorescent MNPs into THP-1-derived macrophages was assessed using a Rescan fluorescence confocal microscope (Amsterdam, The Netherlands). First, THP-1 cells were seeded and differentiated into macrophages as described above (Sect. "Cell culture") in 8-well µ-slides with a tissue culture-treated glass bottom designed for confocal imaging (Ibidi GmbH, Gräfelfing, Germany). The cells were subsequently exposed to 125 µg/ml of serum-coated or digested+serum-coated MNPs. A digestion blank and negative control containing only culture medium and cells were also included. After 1 h of MNP exposure, the cells were washed once with 300 µl of PBS, and the cell surface was stained with 10 ng/ml wheat-germ agglutinin in blocking buffer (2% FBS in PBS) for 1 h. The cells were fixed and washed as described in Sect. "Quantitative measurements of cell association".

### Sodium dodecyl sulfate–polyacrylamide gel electrophoresis

The composition of the protein corona was qualitatively assessed using sodium dodecyl sulfate–polyacrylamide gel electrophoresis (SDS‒PAGE), based on the protocol published by Walczak et al. 2015 [24]. Digested, digested+serum-coated and serum-coated MNPs were separated from the unbound proteins by centrifugation at 30,000 g for 30 min using a tabletop centrifuge. The supernatant was discarded, and the pellet was washed with 1 mL of PBS followed by manual resuspension through pipetting and 10 s of vortexing followed by another centrifugation. The washing step and subsequent centrifugation were repeated twice. Next, the pellet was resuspended in 100 µl of 1X Laemmli sample buffer using a sonication bath followed by vortexing for 10 s, and the samples were subsequently boiled at 95 °C for 5 min. To reduce the amount of MNPs loaded into the SDS‒PAGE gel, MNPs were separated from the protein fraction dissolved in Laemmli buffer by centrifugation at 30,000 g for 5 min. An equal volume of protein of each MNP sample was loaded into a SurePAGE MOPS-Tris 10-well PAGE gel (Genscript EU, Netherlands), and the gel was run at 120 V. Afterwards, the gels were washed twice with demi-water for 10 min to remove excess SDS. The gels were submerged into fixation solution (10% glacial acetic acid, 40% methanol, 50% demi-water) for 20 min followed by a washing step with demi-water for 10 min. The gels were stained using Biosafe G-250 Coomassie for 2 h and were destained with water overnight. An Odyssey gel-imaging system (Li-COR, Homburg vor der Höhe, Germany) was used to capture the fluorescence at 700 nm originating from Coomassie blue, and subsequent images were analyzed using Fiji 2.9.0 as previously published [47].

### Semiquantitative protein corona determination by proteomics

The composition of the protein corona was quantified after in vitro gastrointestinal digestion, serum incubation or the combination using an on-bead protein digestion protocol based on the protocol described by Wendrich et al. (2017) [48]. After in vitro gastrointestinal digestion or incubation in culture medium, the MNPs were centrifuged at 30,000 g for 30 min. The supernatant was discarded, and the pellet was washed with 1 mL of 1 M ammonium bicarbonate buffer followed by 5 s of sonication and 10 s of vortexing. The washing step was repeated once with 1 M ammonium bicarbonate buffer, and afterwards, the pellet was washed with 50 mM of ammonium bicarbonate buffer. The supernatant was removed, and the MNPs were resuspended in 50 µl of 50 mM ammonium bicarbonate buffer. The protein sample was chemically reduced by adding 5 µl of freshly prepared 150 mM dithiothreitol and was incubated for 30 min at 45 °C while shaking at 500 RPM. Five microliters of 200 mM iodoacetamide was added, and the sample was incubated at 20 °C in the dark for 30 min. Six microliters of 200 mM of cysteine in ammonium bicarbonate buffer was added to stop the alkylation by iodoacetamide, followed by the addition of 500 ng of sequencing grade trypsin for an overnight digestion at 25 °C while shaking at 350 RPM. The following day, the enzymatic digestion was stopped by the addition of 3 µl of 10% tri-fluoro acetic acid to the samples. The peptide samples were cleaned using the µ-column method as previously described [49]. The solvent remaining after µ-column sample clean-up was removed using a rotary evaporator, and the peptide samples were redissolved in 50 µl of 1% formic acid in demi water. The samples were subsequently measured using a nano LC‒MS–MS protocol as previously described [23]. Briefly, 5 µl of tryptic peptide solution was injected into a 0.10 × 250 mm ReproSil-Pur 120 C18-AQ 1.9 μm beads analytical column (prepared in-house) at 800 bar. A gradient from 9 to 34% acetonitrile in water with 0.1% formic acid in 50 min (Thermo Vanquish Neo) was used. Full scan FTMS spectra were obtained using an Orbitrap Exploris 480 Thermo electron (San Jose, CA, USA) in positive mode between 380 and 1400 m/z. The 25 most abundant positively charged peaks in the MS scan were fragmented (HCD) with an isolation width of 1.2 m/z and 24% normalized collision energy. Samples were measured in triplicate. Since the in vitro digestion protocol contains proteases in addition to trypsin, nonspecific digestion was assumed. To account for peptides originating from digestion by non-trypsin proteases, an in-house database of nonspecific porcine and bovine peptides was generated by heat inactivation of mixed bovine serum, bile and porcine pancreatin followed by tryptic digestion. The found tryptic peptides were mapped against the bovine and porcine Uniprot database to obtain a minimal library of potential proteins which could be encountered in the sample. Proteins in each sample were identified by comparing the identified peptides to the in-house bovine and porcine databases.

### Analysis of LC‒MS–MS-based proteomics data

The label-free quantification (LFQ) values were obtained from MaxQuant [50] and were analyzed using Perseus [51]. First non-bovine or porcine contaminant proteins, proteins that did not have at least 2 valid values in any condition and proteins only identified by modified peptides (only identified by site) were removed from the dataset. Then, protein annotations, including GO biological process, molecular function, protein family and KEGG function, were retrieved using the Bos Taurus and Sus Scrofa UniProt databases [52]. To visualize proteins present on the PS MNPs upon different treatment conditions, a Venn diagram and upset plot were generated in R using the R Graph gallery VennDiagram [53] and the UpSetR [54] packages. The raw LFQ values were log2 transformed, and missing values were imputed from a normal distribution using a downshift of 3.0 and default band narrowing. The log2 transformed values were used to generate a PCA plot, a heatmap of the Pearson correlation coefficient and a hierarchical clustering of the protein abundance. Differentially abundant proteins were identified using a two-tailed T test with permutation-based false discovery rate (FDR) correction using 250 simulations. Proteins were considered differentially abundant if they had a p value smaller than 0.05 and an absolute log2-fold change larger than 2. The results were plotted as volcano plots using the volcano plot function of Perseus. The correlation between particle uptake and protein abundance was computed in GraphPad 9 by taking the luminescence values obtained from flow cytometry for cells treated with the highest concentration of MNPs and by subsequently using the GraphPad 9 correlation function to correlate this to the averaged protein LFQ value obtained for each of the treatment-particle combinations. Proteins that showed a Pearson correlation coefficient larger than 0.5 were considered correlated, and the resulting correlated proteins were manually compared to previous publications [55, 56] and the annotations obtained from the UniProt database [52]. To assess enrichment of protein functions in differentially abundant proteins and proteins correlated with uptake, a Fischers exact test with Benjamini‒Hochberg-based FDR correction was performed to determine if there was a significant association between the proteins of interest and specific GO terms. KEGG and GO functions with an FDR smaller than 0.05 were considered significantly enriched.

## Results

### In vitro* gastrointestinal digestion does not significantly affect particle size*

To characterize the hydrodynamic sizes of the MNPs upon in vitro gastrointestinal digestion, we used DLS. The hydrodynamic size of pristine, digested+serum-coated and serum-coated MNPs was measured in serum-free culture medium (Table 4).

**Table 4:** Hydrodynamic size distribution of pristine, serum-coated, and digested+serum-coated MNPs

	Pristine MNPs		Serum-coated MNPs		Digested+serum-coated MNPs	
MNP	z-avg ± SD [nm]	PDI ± SD	z-avg ± SD [nm]	PDI ± SD	z-avg ± SD [nm]	PDI ± SD
PS50	84 ± 13	0.08 ± 0.05	117 ± 9	0.17 ± 0.01	285 ± 55	0.52 ± 0.11*
PS100	109 ± 1	0.02 ± 0.01	142 ± 4	0.08 ± 0.01*	165 ± 32	0.17 ± 0.08
PS100 (−)	106 ± 1	0.04 ± 0.01	160 ± 8	0.16 ± 0.03	255 ± 27	0.26 ± 0.10
PS100 (+)	1494 ± 214	0.24 ± 0.04	608 ± 6*	0.73 ± 0.29	1630 ± 304	0.63 ± 0.35
PS200	191 ± 1	0.02 ± 0.01	221 ± 2	0.03 ± 0.01	234 ± 2	0.09 ± 0.01*
PS500	561 ± 51	0.07 ± 0.04	544 ± 2	0.17 ± 0.02	531 ± 11	0.17 ± 0.02
PS1000	1160 ± 143	0.10 ± 0.04	1038 ± 61	0.27 ± 0.10	1009 ± 41	0.28 ± 0.13

The sizes are the z-average hydrodynamic size of each of the MNPs and the polydispersity index (PDI). The particle size of pristine MNPs was measured in serum-free RPMI 1640 culture medium. The sizes and PDIs shown are the average of 3 replicates, each consisting of 5 measurements. A two-way ANOVA with Bonferroni post hoc correction was performed to assess significant differences from the pristine particles. Mean ± SD, n = 3, * indicates P < 0.05.

While an increase in the average sizes of MNPs was observed upon digestion, these increases were not statistically significant. The hydrodynamic size of the PS100 (+) MNPs was larger than the ‘nominal’ sizes (i.e., 100 nm) in all incubation conditions. The only significant incubation-related difference in average hydrodynamic MNP size was found for serum-coated PS100 (+), potentially due to the stabilization of MNPs by surrounding proteins.

### *Viability of THP-1-derived macrophages was unaffected by serum-coated or digested*+*serum-coated MNPs*

**Fig. 1:**
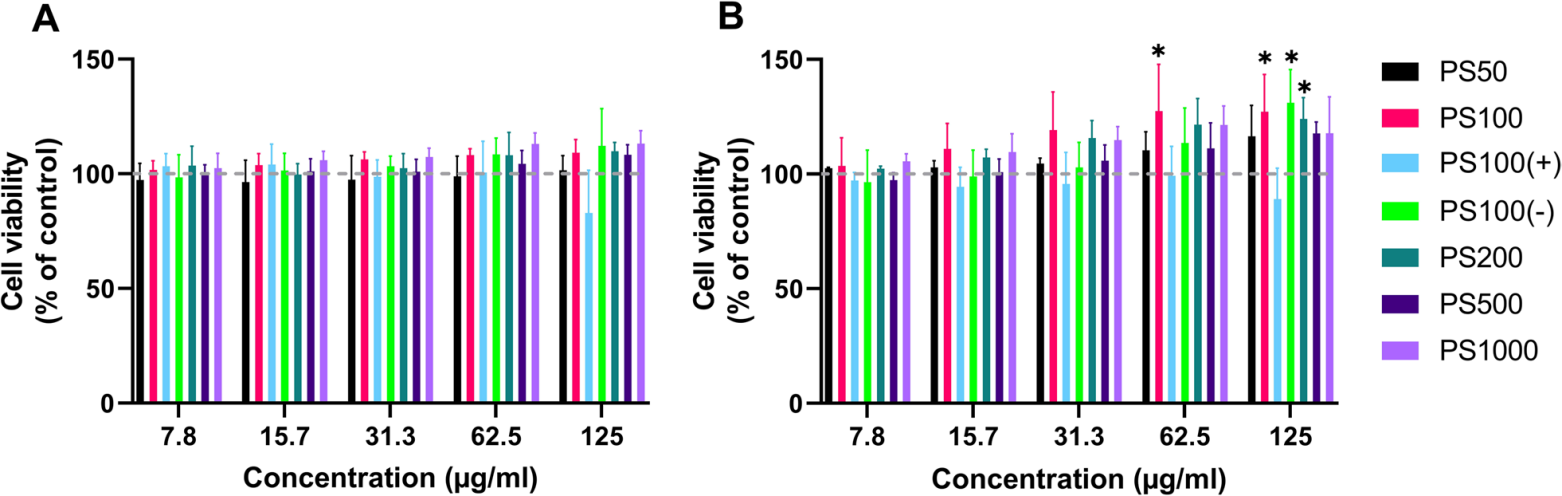
Effect of digested+serum-coated or serum-coated MNPs on THP-1 cell viability. **A** Cell viability after 24 h of exposure to serum-coated MNPs. **B** Cell viability after 24 h of exposure to digested+serum-coated-MNPs. Viability is expressed as the relative absorption at 440 nm compared to the negative control. A two-way ANOVA with Bonferroni post hoc correction was performed to assess significant differences from the medium control. Mean ± SD, n = 3, * indicates P < 0.05

A WST-1 assay was performed to assess the viability of THP-1-derived macrophages upon exposure to differently treated MNP (Fig. 1).

Exposure of THP-1-derived macrophages to serum-coated MNPs did not significantly affect the viability of the cells (Fig. 1A) at any of the tested concentrations. Incubation of the THP-1-derived macrophages for 24 h with digested+serum-coated MNPs also did not reduce the cell viability for any of the MNPs (Fig. 1B), although an increase in mitochondrial activity was observed after exposure to digested+serum-coated PS100, PS100 (−) PS100 (+) MNPs at 62.5 and 125 µg/ml. Based on these results, for the MNP cell association and uptake studies, a maximal MNP concentration of 125 µg/ml was used. Predictions obtained using the ISDD model indicated that the fractions deposited after 24 h of incubation for the PS50 MNPs were 77.6% and 58.1% for PS100, 45% for PS200, 63% for PS500 and 100% for PS1000 (Additional file 1: Fig. S6). The influence of the charge of the PS100 (−) and PS100 (+) MNP on sedimentation was not considered in the ISDD model.

### In vitro* gastrointestinal digestion increases the cell association of small but not of large or charged MNPs*

**Fig. 2:**
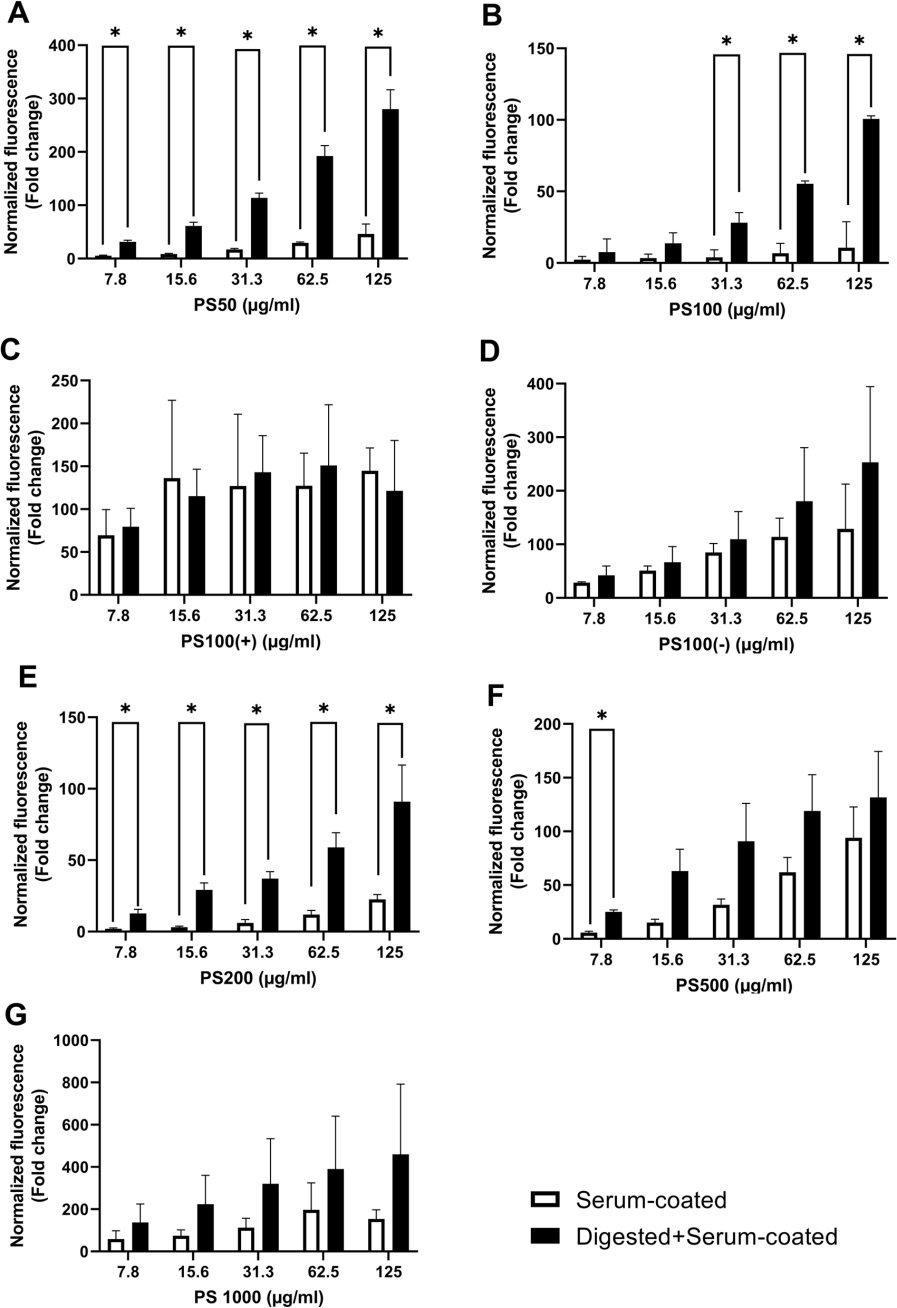
Concentration-dependent THP-1-derived macrophage association of MNPs. MNP cell association after 24 h of exposure to 7.8, 15.6, 31.3, 62.5 or 125 µg/ml of serum-coated or digested+serum-coated MNPs. The graphs show the cell association of serum-coated (white bars) or digested+serum-coated (black bars) **A** PS50, **B** PS100, **C** PS100 (+), **D** PS100 (−), **E** PS200, **F** PS500 and **G** PS1000 MNPs. The y-axis shows the fold change increase in fluorescence compared to untreated cells that has been corrected to reflect the difference in fluorescence per µg of MNPs as determined by fluorimetry (Additional file 1: Fig. S5). A two-way ANOVA with Bonferroni post hoc correction was performed to assess significant differences from the medium control. Mean ± SD of n = 3, * indicates P < 0.05

The cell association and uptake of PS MNPs by THP-1-derived macrophages exposed to serum-coated and digested+serum-coated PS MNPs were assessed by flow cytometry (Fig. 2). For PS50, PS100 and PS200, the cell association was significantly higher at all concentrations for the digested+serum-coated MNPs compared to the serum-coated MNPs, being 6.1, 6.0 and 4.0 times higher for the highest concentration of PS50, PS100 and PS200, respectively. For PS500, a similar trend was observed, but significance was only reached at the lowest concentration used. No significant differences between serum-coated and digested+serum-coated MNP cell associations were observed for the PS1000, PS100 (−) and PS100 (+) MNPs.

### *Both digested*+*serum-coated and serum-coated PS MNPs are rapidly internalized by THP-1-derived macrophages.*

**Fig. 3:**
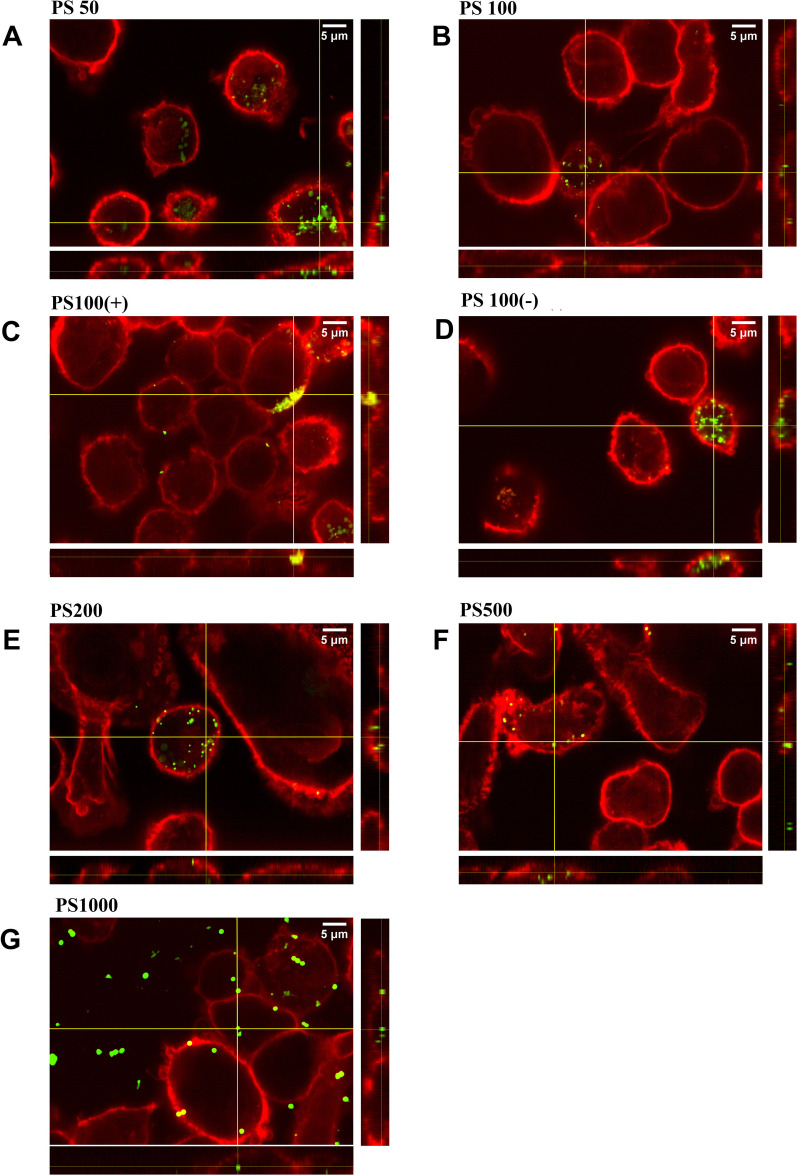
Internalization of serum-coated MNPs by THP-1-derived macrophages after 1 h of exposure. Cells were exposed to 125 µg/ml of serum-coated MNPs **A** PS50, **B** PS100, **C** PS100 (+), **D** PS100 (−), **E** PS200, **F** PS500 and **g** PS1000. At the side of each image, the orthogonal Y–Z and at the bottom, the orthogonal X–Z views are shown, which represent slices through the Y–Z and X–Z planes. The yellow cross in the X–Y image indicates the origin of the orthogonal images. The red stain corresponds to the wheat germ agglutinin cell surface coating, and the green stain corresponds to the fluorescent PS MNPs

**Fig. 4:**
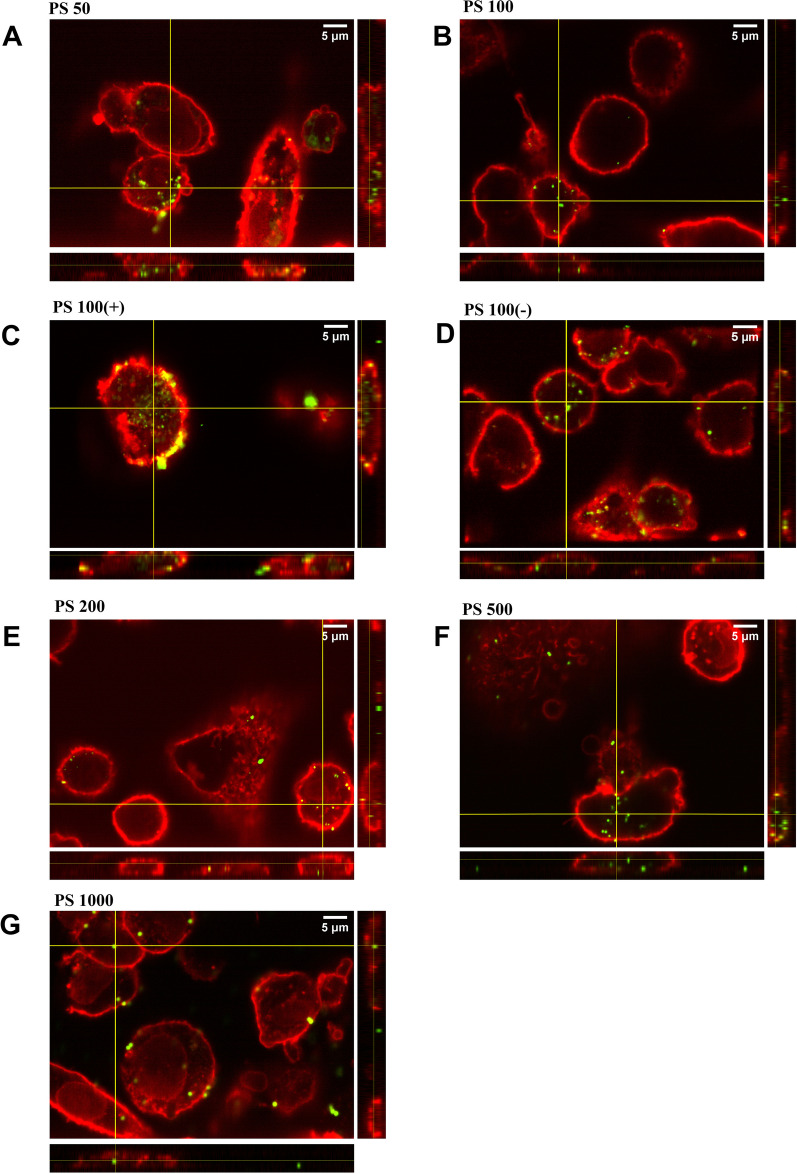
Internalization of digested+serum-coated MNPs by THP-1-derived macrophages after 1 h of exposure. Cells were exposed to 125 µg/ml of digested+serum-coated or MNPs **A** serum-coated PS50, **B** PS100, **C** PS100–Z and at the bottom, the orthogonal X–Z views are shown, which represent slices through the Y–Z and X–Z planes. The yellow cross the X–Y image indicates the origin of the orthogonal images. The red stain corresponds to the wheat germ agglutinin cell surface coating, and the green stain corresponds to the fluorescent PS MNPs

To differentiate between cell association and cellular uptake, THP-1-derived macrophages were exposed to serum-coated MNPs (Fig. 3) or digested+serum-coated MNPs (Fig. 4). Cell membranes were stained with wheat germ agglutinin, and the MNP-exposed cells were analyzed using confocal microscopy.

For all types of PS MNPs, we observed cell internalization within 1 h of exposure to digested+serum-coated MNPs or serum-coated MNPs. Some cell surface-bound MNPs can be observed for the digested+serum-coated PS100 (+) and PS1000 MNPs, but overall, the majority of the MNPs are localized intracellularly. The washing steps removed nearly all of the unbound MNPs, allowing us to conclude that the flow cytometry results can be interpreted as MNP cellular uptake rather than (only) membrane association.

### The digestion corona is retained upon serum incubation and affects the subsequent binding of serum proteins

**Fig. 5:**
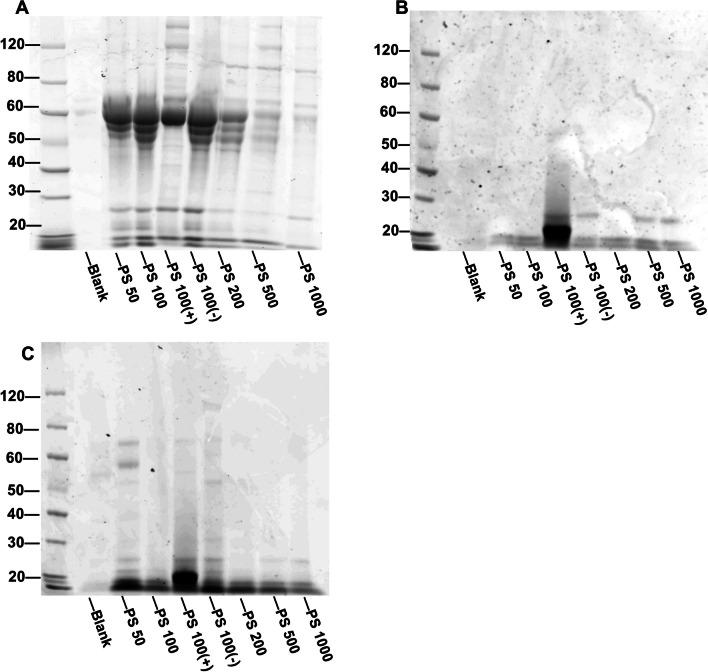
Protein corona composition of MNPs with different incubation histories. **A** SDS‒PAGE of proteins isolated from serum-coated MNPs. **B** SDS‒PAGE of proteins isolated from in vitro intestinal digested MNPs. **C** SDS‒PAGE of proteins isolated from digested+serum-coated MNPs. The protein sizes in kDa are shown to the left of each image. The samples corresponding to each lane are shown at the bottom of each image. Blank = H_2_O addition instead of MNP addition. Representative image of n = 3

For the assessment of the biomolecular corona on the MNPs, the protein composition was first assessed using SDS‒PAGE (Fig. 5A–C). Upon incubation of the MNPs in serum containing medium (Fig. 5A), a high intensity and broad band between 50 and 60 kDa was observed for most MNPs except for the PS500 and PS1000 MNPs, which showed low overall protein content of their corona.

Upon in vitro gastrointestinal digestion (Fig. 5B), the different MNPs showed little variation in the protein bands observed on the gel, which largely corresponded to proteins and peptides smaller than 30 kDa. The only exception is for PS100 (+), which shows a smear of proteins and peptides ranging between 15 and 60 kDa and a much more pronounced band at 20 kDa.

Lastly, MNPs were transferred from the in vitro gastrointestinal digestion matrix into complete cell culture medium, which represents the exposure conditions used in the viability and uptake studies (Fig. 5C). The resulting digested+serum-coated MNP corona was loaded on the gel. Most of the bands observed for the MNPs are similar to the patterns observed in the digestion corona, indicating that the digestion-associated corona is retained in high serum conditions. PS50 and the PS100 (+) MNPs show faint retention of serum protein. When comparing Fig. 5B–C, it can be seen that the serum protein pattern was greatly reduced by the presence of the digestion-associated corona.

### Proteomic analysis of the serum and digested+serum-coated protein corona on PS MNPs

LC‒MS–MS-based proteomics was performed to investigate potential treatment-related alterations in the protein corona on a subset of PS MNPs. From the MNPs for which in vitro digestion significantly affected uptake, we selected the PS100 MNPs. To study the influence of the charge of MNPs, we included PS100 (+) and PS100 (−) MNPs. Lastly, the PS1000 MNPs were included in the corona proteomics analysis to represent larger PS MNPs.

First, the global protein similarity between samples was assessed. Then, all proteins that showed a significant increase in abundance between treatment types were identified. Finally, the Pearson correlation coefficient between protein abundance and particle uptake at 125 µg/ml (based on flow cytometry) was calculated.

#### Global overview of the PS MNP protein corona

In total, 280 proteins were identified in the corona of the PS MNPs, of which 219 proteins were found on serum-coated PS MNPs and 173 proteins were found on the digested+serum-coated PS MNPs (Table 5).

**Table 5:** Number of proteins identified during semiquantitative LC‒MS–MS proteomics

Particle	Proteins on serum-coated MNPs	Proteins on digested+serum-coated MNPs
Total	219	173
PS100	169	105
PS100 (+)	192	157
PS100 (−)	167	113
PS1000	191	125

**Fig. 6:**
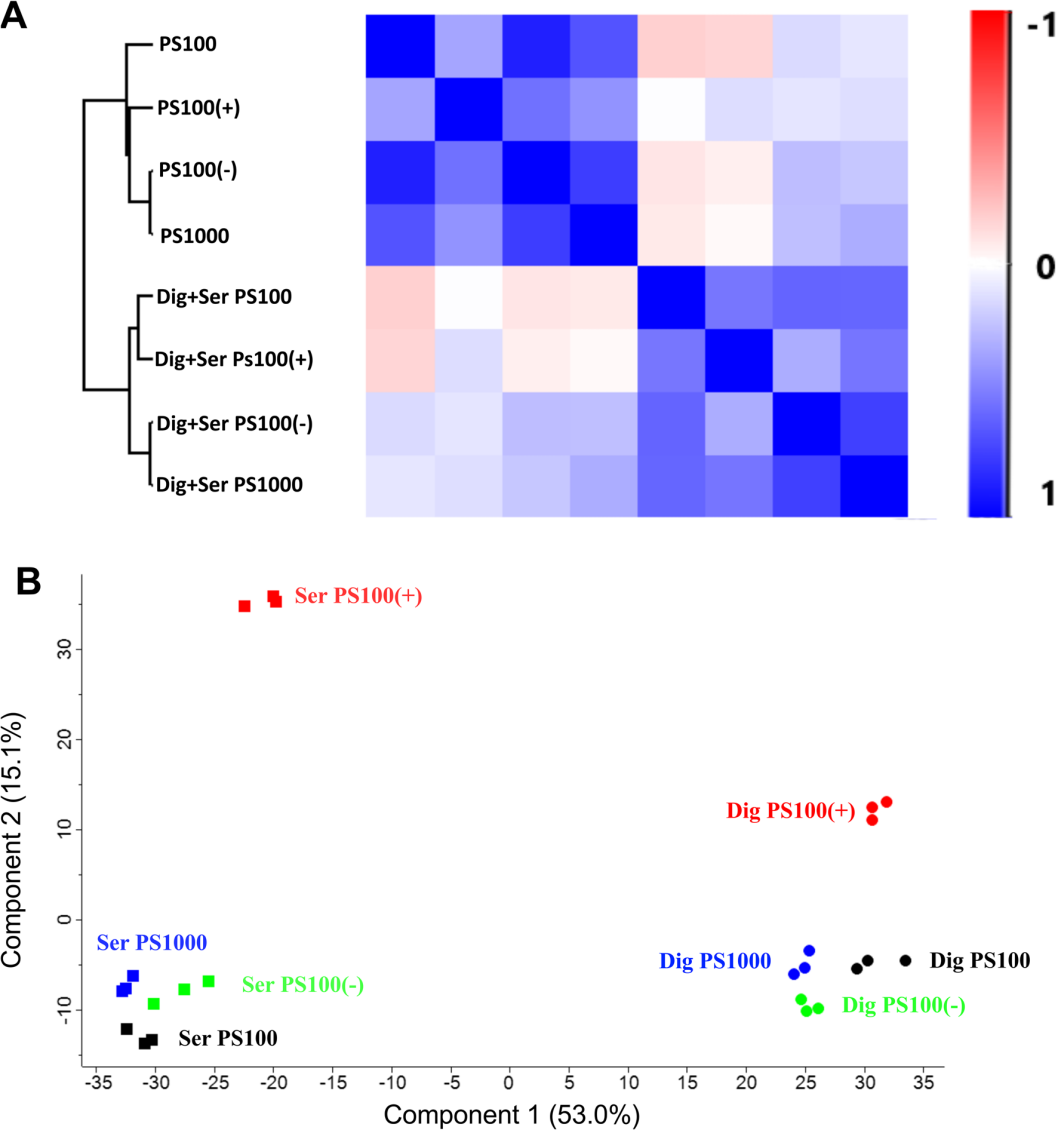
Similarity between the protein coronas identified on MNPs. **A** Heatmap indicating the Pearson correlation coefficient between the median amount of all identified proteins identified on each PS MNP type. The dendrogram shown to the left of the heatmap indicates hierarchical clustering of the different MNPs, and the X- and Y-axes indicate the treatment-particle combination. The bar to the right of the heatmap indicates the Pearson correlation coefficient corresponding to each color. In the legend on the X- and Y-axes, Ser refers to serum-coated particles, while DIG+Ser refers to digested+serum-coated particles. **B** Principal component analysis of proteomics samples. Filled squares represent serum-coated MNPs, and filled circles represent digested+serum-coated MNPs. Each symbol represents an LC‒MS–MS sample. The color of each symbol indicates the MNP type, with black corresponding to PS100, red corresponding to PS100 (+), green corresponding to PS100 (−) and blue corresponding to PS1000 MNPs. (n = 3)

**Fig. 7:**
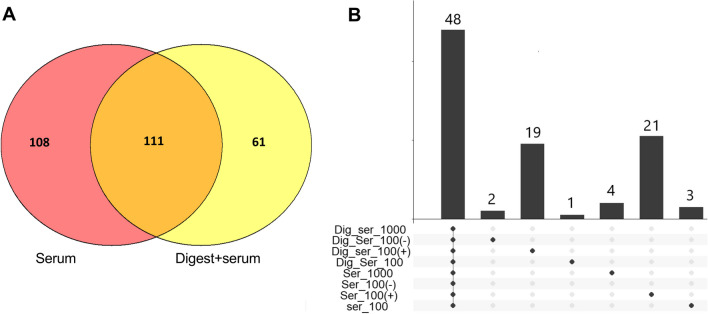
Number of identified proteins present in the protein corona on PS MNPs. **A** Venn diagram illustrating overlap between proteins identified on serum-coated and digested+serum-coated PS MNPs. The numbers in each circle indicate the number of unique proteins identified. The area where both circles overlap indicates proteins identified in both serum-coated and digested+serum-coated PS MNPs. **B** An upset plot indicating the number of proteins found on all types of PS MNPs and the number of proteins found only in one of the treated MNPs. The dots indicate the combinations of MNPs, while the bar chart on top shows the number of proteins identified in this respective group. Serum-coated PS100 (−) and digested+serum-coated PS1000 MNPs had no unique proteins and are not shown as individual columns in the upset plot

Similarity between samples was assessed by computing the Pearson correlation coefficient between inter-sample protein abundances (Fig. 6A) and by performing a principal component analysis (Fig. 6B). Then, proteins unique to any of the particle-treatment combinations were identified and visualized using a Venn diagram (Fig. 7A) and UpSet plot (Fig. 7B).

As shown in Fig. 6A, the serum-coated and digested+serum-coated PS MNPs showed a high correlation within but a low correlation between treatment groups. Of the digested+serum-coated MNPs, the PS100 MNPs were most different from the serum-coated MNPs (average correlation − 0.06), while the PS100 (−) MNPs showed the highest average correlation of 0.19. The PS100 (+) MNPs showed the highest correlation with the digested+serum-coated MNPs (0.09). Interestingly, the serum-coated PS100 MNPs showed the lowest correlation with the digested+serum-coated MNPs (− 0.18), indicating a larger impact of digestion compared to the other MNPs.

The principal component analysis revealed that 53.0% of all variation in protein abundances could be explained by the treatment. The second largest source of variation comprising 15.1% was linked to the presence or absence of a positive charge.

We next assessed which proteins were unique to the serum corona or digested+serum corona (Fig. 7).

Of the 280 proteins that we identified in the MNP corona, 108 were found only in the corona of serum-coated MNPs, 62 were found only in the digested+serum corona, and 111 proteins were shared among the two types of MNP coronas (Fig. 7A). Looking in more detail, 48 proteins were found to be present on all MNPs (Fig. 7B). The PS100 (+) MNPs contained the highest number of unique proteins, 21 in the serum-coated and 19 unique proteins on the digested+serum-coated PS100 (+) MNPs. The proteins that were unique to any of the particle treatment combinations are listed in Additional file 1: Table 1.

### Identifying differentially abundant proteins in the corona on PS MNPs

**Fig. 8:**
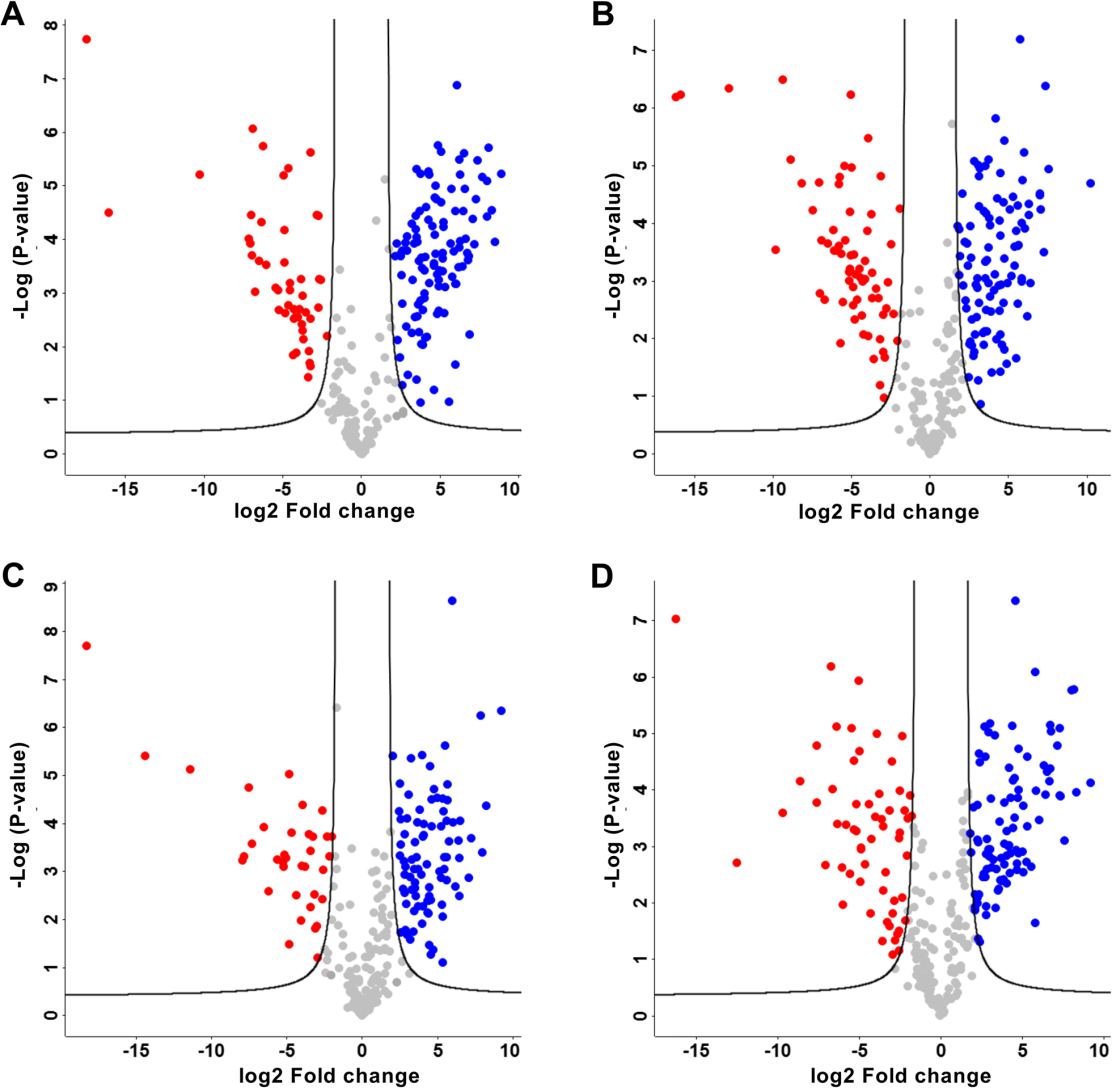
Differential abundance of proteins between serum-coated and digested+serum-coated MNPs. Differential abundance of proteins in serum-coated vs. digested+serum-coated PS MNPs **A** PS100, **B** PS100 (+), **C** PS100 (−), **D** PS1000 MNPs. Every dot represents a unique protein identified. The X-axis represents the log2 (fold change) between the serum-coated and digested+serum-coated MNPs. The y-axis indicates the -log of the P value. The black lines indicate the P value threshold and fold-change threshold used to consider a protein as differentially abundant. Colored dots are differentially abundant, while dots in gray are considered equal between treatments. A negative log2-fold change (red dots) represents proteins that are more abundant in the digested+serum-coated protein corona, while a positive log2-fold change (blue dots) represents proteins more abundant in the serum-coated protein corona. Differential expression testing was done by multiple T-tests with permutation based FDR correction at FDR = 0.05. Proteins with an absolute log2fold difference larger than 2 and a P-value below 0.05 were considered significantly differentially abundant

To assess which proteins were differentially abundant on the different MNPs, a differential expression analysis of the samples was performed using a two-tailed T test with permutation-based FDR correction (Fig. 8 and Additional file 2).

The differential expression analysis showed 166, 174, 134 and 163 differentially abundant proteins on PS100, PS100 ( +), PS100 (−) and PS1000 MNPs, respectively. Of these, 48, 62, 42 and 51 proteins were significantly more abunFdant in the digested+serum-coated PS MNPs, while 118, 112, 92 and 112 proteins were significantly more abundant in the serum corona on PS100, PS100 (+), PS100 (−) and PS1000 MNPs, respectively (Fig. 8, and Supplementary file S1). A protein set enrichment analysis showed that the overrepresented proteins in the serum-coated PS100 corona were significantly enriched for proteins involved in the complement and coagulation cascades, while the remaining serum-coated particles had no significant enrichment of KEGG or GO functions. Proteins overrepresented in the digested+serum corona of PS MNP had no specific functional protein set enrichment (Additional file 2). Proteins that were differentially abundant in one of the particle treatment combinations are shown in Additional file 1: Table S1. For PS100, PS100 (+), PS100 (−) and PS1000, 15, 29, one and nine proteins were found to be uniquely more abundant in serum-coated conditions, and five, 21, three and one proteins were uniquely more abundant in the digested+serum-coated conditions (Fig. 8 & Table 6).

**Table 6:** Proteins uniquely differentially present in a specific PS MNP corona

Condition	Unique differentially abundant proteins		
Serum-coated PS 100	F1N045 (Complement C7)	Q3MHL4 (Adenosylhomocysteinase)	F1MB08 (phosphopyruvate hydratase)
	F1MW44(coagulation factor 13 chain)	Q2HJ57 (Coactosin like protein)	Q27967 (Secreted phosphoprotein 24)
	Q2KJ83 (carboxypeptidase N-catalytic chain)	G3MZI7 (Collagen type V alpha chain)	P80012 (von willebrand factor)
	Q28085 (complement factor H)	P17697 (Clusterin)	Q0VCM5 (ITIH 1)
	P00735 (Prothrombin)	A5D7R6 (ITIH-2)	A0A3Q1NJR8 (Antithrombin-3)
Serum-coated PS100 (+)	A0A0A0MP92 (Endopin 2)	E1BF81 (Corticosteroid binding globulin)	A0A0A0MPA0 (Serpin domain containing protein)
	P13605 (Fibromodulin)	F6RMV5 (Leucine rich alpha-2-glycoprotein 1)	Q9TT36 (Thyoxine-binding globulin)
	P01267 (Thyroglobulin)	F1MPD1 (Mannose receptor C type 2)	A6QPQ2 (Serpin A3)
	P01017 (Angiotensinogen)	O77742 (Osteomodulin)	I2E4T6 (MBL assosciated serine protease 2)
	Q09TE3 (ILGF-binding protein labile subunit)	Q95M17 (Acidic mamalian chitinase)	A0A287BDF8 (Inositol-3-phosphate synthase 1)
	F1MPE1 (CD109)	F1RU49 (Actinin alpha 3)	F1MJQ3 (alpha amylase)
	A7YWG4 (gamma-glutamyl hydrolase)	E1BH94 (peptidoglycan recognicion protein 2)	Q58CQ9 (Pantetheinase)
	F1MVK1 (uncharacterized protein)	A5PKC2 (SHBG protein)	A0A287AY54 (60S acidic ribosomal protein)
	G5E5T5 (Ig-like domain containing protein)	A5PK77 (Serpin A11 protein)	P56652 (ITIH-3)
	F1MMD7 (ITIH-4)	F1MJK3 (Pregnancy zone protein)	–
Serum-coated PS100 (−)	A0A287AFA5 (Ras homolog member F)	–	–
Serum-coated PS 1000	P33072 (Protein-lysine-6-oxidase)	P35445 (Cartilage oligomeric matrix protein)	F1SHM0 (pyruvate kinase M1/2)
	Q2KIT0 (Protein HP20-homolog)	P19879 (Mimecan)	I3LQ84 (Collagen type 6 alpha 2 chain)
	P07224 (Vitamin K dependent protein S)	Q5E946 (Parkinson disease protein 7 homolog)	F1N0I3 (Coagulation factor 5)
Digested+serum-coated PS 100	F1N5M2 (Vitamin D binding protein)	Q32KY0 (apolipoprotein D)	A0A3Q1MFR4 (Apolipoprotein B)
	P15497 (Apolipoprotein A-1)	A0A3Q1M1Z4 (Ig-like domain containing protein)	–
Digested+serum-coated PS 100(+)	E1B9F6 (Elongation factor 2-alpha)	F1SML4 (SND-domain containing) protein)	Q29375 (Large ribosomal subunit eL8)
	A0A5G2QSB4 (ribosomal protein S3)	P01846 (Ig lambda chain C region)	K7GLN4 (Peroxyredoxin 4)
	F2Z4Y8 (40S ribosomal protein S11)	P28839 (cytosol aminopeptidase)	Q29036 (DAD-1)
	A0A5G2Q9S0 (Nucleoside diphosphatase kinase)	F1SQT3 (mitochondrial phosphate carrier protein)	A0A287A014 (40S ribosomal protein S15a)
	A0A5G2R5F8 (KDEL endoplasmic reticulum protein retention receptor)	F1SQW6 (endoplasmic reticulum protein 27)	P80021 (ATP synthase mitochondrial subunit)
	A0A286ZKG9 (Peptidyl-prolyl cis–trans isomerase)	A0A286ZW72 (60S ribosomal protein L14)	P26779 (Proaposin)
	K7GNY4 (60S ribosomal protein L8)	F1RW28 (hydroxysteroid 17-beta dehydrogenase)	F1SFA7 (collagen type 1 alpha 2 chain)
Digested+serum-coated PS 100(−)	A0A287AYH9 (Palmtioyl-protein thioesterase 1)	F1MZ96 (Ig-like domain containing protein)	G3MYZ3 (Afamin)
Digested+serum-coated PS 1000	G3N0S9 (Sushi domain containing protein)	–	–

This table highlights differentially abundant proteins which were only enriched on one single particle treatment combination. The leftmost column shows the corresponding particle and treatment while the 3 rightmost columns indicate the Uniprot identified and full name of the identified protein

### Correlation of corona proteins with cellular uptake of PS MNPs

**Fig. 9:**
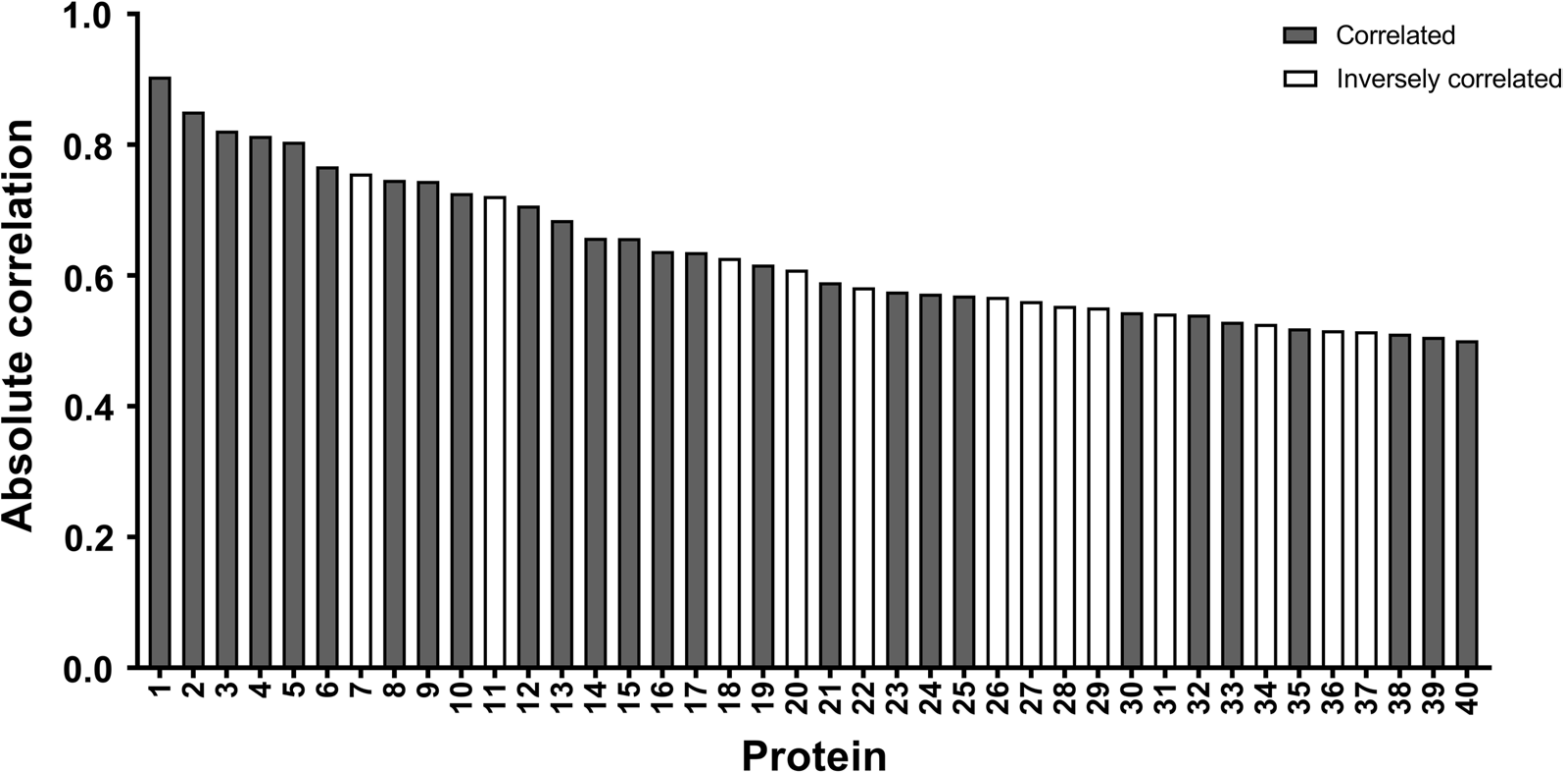
Overview of proteins present in a PS MNP corona that correlated with particle uptake. Pearson correlation coefficient of the protein abundance with MNP uptake of the 40 most correlated proteins. Correlated proteins were defined as having an absolute correlation if the coefficient > 0.5. MNP uptake was determined using flow cytometry and was defined as the average relative light unit per treatment condition. Protein abundance in the MNP corona was obtained using LC‒MS–MS and was defined as the average LFQ of the triplicate samples. The numbers shown on the x-axis refer to the proteins listed in Table 7. The y-axis shows the Pearson correlation coefficient. A positive coefficient indicates that the protein is correlated with uptake, while a negative correlation coefficient indicates that the protein is inversely correlated with uptake. The color (black or white) indicates whether the protein is correlated or inversely correlated (p < 0.05, see Table 7)

To verify whether the proteins identified in the differential expression analysis contribute to differences in the observed uptake by THP-1-derived macrophages, the Pearson correlation coefficient was calculated for the relation between the protein abundances identified through LC‒MS–MS and the uptake in relative light units identified through flow cytometry. Figure 9 and Table 7 show the Pearson correlation coefficient of the 40 proteins most correlated with the uptake of PS MNPs. In total, 40 proteins were identified that had a correlation larger than 0.5 with uptake; for 12 proteins, this correlation was statistically significant (Fig. 9 and Table 7). A graphical representation of the protein correlation is also shown in the Additional file 1: Fig. S7).

**Table 7:** Proteins showing a significant correlation with PS MNP uptake

Protein ID	Protein name	R-coefficient	P value	upregulated in	Fig. 9 label
P81644	Apolipoprotein A-II	0.90	0.002	Digested+serum-coated PS100 Digested+serum-coated PS100 (−) Serum-coated PS100 (+)	1
A0A3Q1MGI2	Apolipoprotein E	0.85	0.007	–	2
P19034	Apolipoprotein C-II	0.82	0.012	Serum-coated PS100 (+) Serum-coated PS1000	3
P02081	Hemoglobin fetal subunit beta	0.81	0.014	–	4
P02639	Protein S100-A1	0.80	0.015	Serum-coated PS100 (+) Serum-coated PS1000	5
A0A5G2QL09	S-adenosylmethionine synthase	0.77	0.026	Serum-coated PS100 (+) Serum-coated PS1000	6
G3MZI7	Collagen type V alpha 1 chain	− 0.76	0.030	Serum-coated PS100	7
P01965/P01966	Hemoglobin subunit alpha	0.75	0.033	All	8
P62833	Ras-related protein Rap-1A	0.75	0.034	–	9
Q3ZBS7	Vitronectin	0.73	0.041	–	10
Q58D62	Fetuin-B	− 0.72	0.043	–	11
P19879	Mimecan	0.71	0.049	Serum-coated PS1000	12
Q3MHN5	Vitamin D-binding protein	0.68	0.061	Digested+serum-coated PS100 Digested+serum-coated PS100 (+)	13
Q28035	Glutathione S-transferase A1	0.66	0.076	Serum-coated PS100 (+) Serum-coated PS1000	14
F1SHM0	pyruvate kinase M1/2	0.66	0.076	Serum-coated PS1000	15
A0A286ZNV2	Elongation factor 1-alpha 2	0.64	0.089	Digested+serum-coated PS100 Digested+serum-coated PS100 (+)	16
P33072	Protein-lysine 6-oxidase	0.64	0.090	Serum-coated PS1000	17
F1N1W7	Neural cell adhesion molecule 1	− 0.63	0.096	Serum-coated PS100 Serum-coated PS100 (+) Serum-coated PS100 (−)	18
P01035	Cystatin-C	0.62	0.103	Serum-coated PS100 (−) Serum-coated PS1000	19
Q9TTE1	Serpin A3-1	− 0.61	0.109	–	20
P15497	Apolipoprotein A-I	0.59	0.124	Digested+serum-coated PS100	21
P07224	Vitamin K-dependent protein S	− 0.58	0.130	Serum-coated PS1000	22
A0A287AK65	Argininosuccinate synthase	0.58	0.136	Serum-coated PS100 (+) Serum-coated PS1000	23
F1N0I3	Coagulation factor V	0.57	0.138	Serum-coated PS1000	24
F1N102	Complement C8 beta chain	0.57	0.140	All serum-coated MNPs	25
G5E513	Ig-like domain-containing protein	− 0.57	0.142	–	26
Q3SZV7	Hemopexin	− 0.56	0.148	–	27
A0A3Q1M1Z4	Ig-like domain-containing protein	− 0.55	0.155	Digested+serum-coated PS100	28
P80012	von Willebrand factor	− 0.55	0.157	Serum-coated PS100	29
P10096	Glyceraldehyde-3-phosphate dehydrogenase	0.54	0.163	–	30
A0A3Q1M3L6	Ig-like domain-containing protein	− 0.54	0.165	Serum-coated PS100, Serum-coated PS100 (−) Serum-coated PS1000	31
Q5E946	Parkinson disease protein 7 homolog	0.54	0.167	Serum-coated PS1000	32
Q2KIT0	Protein HP-20 homolog	0.53	0.177	Serum-coated PS1000	33
F1SUP2	Signal peptidase complex subunit 2	− 0.53	0.180	Digested+serum-coated PS100 Digested+serum-coated PS100 (+)	34
P00735	Prothrombin	0.52	0.187	Serum-coated PS100	35
E1B805	Complement C3	− 0.52	0.190	Serum-coated PS100 Serum-coated PS100 (−) Serum-coated PS1000	36
A0A3Q1MFR4	Apolipoprotein B	− 0.51	0.191	Digested+serum-coated PS100	37
P81947	Tubulin alpha-1B chain	0.51	0.195	–	38
F1MW44	Coagulation factor XIII A chain	0.51	0.200	Serum-coated PS100	39
P02676	Fibrinogen beta chain	0.50	0.206	Serum-coated PS100 Serum-coated PS100 (+)	40

The columns show the UniProt protein IDs and protein names of all of the proteins that were found to be correlated with MNP uptake. Correlation was defined as having an absolute Pearson correlation coefficient (R) > 0.5, with a positive R-coefficient indicating a positive correlation and a negative R-coefficient indicating an inverse correlation. The table is ordered from highest absolute correlation to lowest absolute correlation. The enriched in the column indicates whether the protein was enriched in any of the particle treatment combinations (Additional file 2). The numbers in the last column provide a link to the x-axis label in Fig. 9

**Fig. 10:**
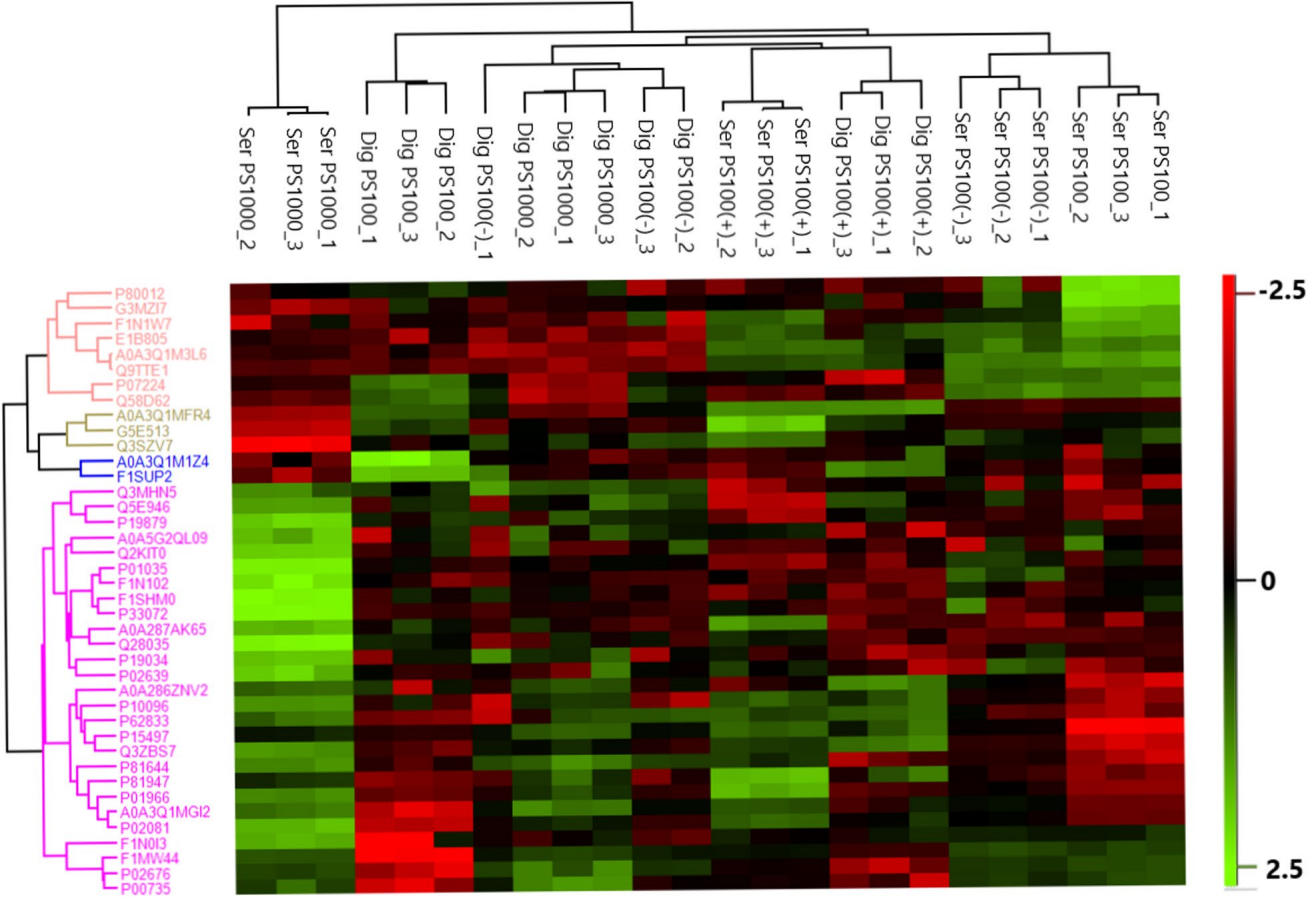
LFQ profiles of the 40 most correlated proteins in the protein corona. Heatmap indicating the normalized log LFQ value of all the proteins correlated with uptake. The dendrogram to the top of the heatmap shows hierarchical clustering of each sample based on the Euclidian distance between protein abundance. The dendrogram to the left of the heatmap indicates hierarchical clustering of the 40 identified proteins correlated with uptake. The bar to the right of the histogram indicates the normalized log LFQ value that corresponds to each color in the heatmap. Missing values were imputed from a normal distribution

To visualize the abundance distribution of the correlated proteins, a hierarchical clustering of the samples based on the protein abundance of the 40 correlated proteins is shown in Fig. 10. Most of the correlated proteins were enriched in the serum-coated conditions, specifically the serum-coated PS100 or PS1000 MNPs. Four major protein clusters can be identified, of which the orange cluster is most abundant in serum-coated PS100, the blue cluster is most abundant in digested+serum-coated PS100, the yellow cluster is highly abundant in serum-coated PS100 (+) and low abundance in serum-coated PS1000 and finally a pink cluster which is the most abundant in PS1000. Both proteins in the blue cluster are inversely correlated with uptake. Based on the clustering distances between serum-coated and digested+serum-coated MNPs, the charged PS MNPs show the most similarity in abundance patterns of proteins correlated with uptake between treatments, while the PS1000 MNPs and the PS100 MNPs show the most differences.

Of the correlated proteins, 30 proteins were differentially abundant in at least one particle (Table 7 and Fig. 10). Proteins enriched in digested+serum-coated PS100 included apolipoprotein A-II, vitamin D-binding protein, elongation factor 1-alpha 2, apolipoprotein A-I and Ig-like domain-containing protein, while serum-coated PS100 was enriched in fetuin-b, neural cell adhesion molecule 1, von Willebrand factor and collagen type V alpha chain. These proteins showed good correlation with the different uptake patterns observed for serum-coated and digested+serum-coated PS100 MNPs. The remaining significantly correlated proteins had a similar protein abundance between serum-coated and digested+serum-coated PS100 MNPs or did not fit well with the observed differences between the uptake of digested+serum-coated and serum-coated MNPs.

## Discussion

The protein corona of smaller MNPs has been recognized as an important determinant of cellular interactions.

The aim of this paper was to identify whether in vitro gastrointestinal digestion of MNPs affects their uptake into THP-1-derived macrophages and whether uptake differences could be explained by the protein corona composition. Here, we found that in vitro gastrointestinal digestion led to an increased uptake of PS50, PS100 and PS200, while in vitro gastrointestinal digestion had little effect on the uptake of PS500 and PS1000 MNPs and did not affect the uptake of PS100 (+) and PS100 (−) MNPs. LC‒MS–MS-based proteomics showed that complement and coagulation proteins and transporter proteins such as apolipoproteins, hemoglobin, fetuin and ITIH2 proteins were differentially abundant on PS MNPs that were either digested in vitro* by* gastrointestinal digestion or only incubated in serum. A Pearson correlation coefficient analysis confirmed that the presence of 40 proteins correlated with cellular uptake, including the proteins mentioned above. The data presented here represent the first report that the digestion-associated protein corona is retained on PS MNPs of different sizes and charges and consequently affects the incorporation of serum proteins in the PS MNP corona, modifying PS MNP cellular uptake in a particle size- and charge-dependent manner.

First, we determined the hydrodynamic sizes of the PS MNPs in the different treatment conditions. While the z-average hydrodynamic sizes of the pristine and digested PS MNPs suspended in serum-containing medium were seemingly larger, these differences were not significant, with the exception of the reduced hydrodynamic sizes of the PS100 (+) MNPs suspended in serum-containing medium. We also confirmed that no fluorescence was leaking from the PS MNPs during any of the treatments, which allows interpretation of fluorescence signals obtained from the flow cytometry and confocal microscopy experiments as coming from the PS MNPs. The cellular association of MNPs cannot easily be separated from cellular uptake using flow cytometry [57]; thus, confocal microscopy analysis was used to confirm the cellular uptake of PS MNPs by THP-1-derived macrophages. All particles except serum-coated and digested+serum-coated PS100+MNPs showed a high degree of internalization after one hour, allowing interpretation of an increased cellular fluorescence as increased PS MNP uptake.

The uptake of uncharged MNPs with sizes < 500 nm was significantly increased upon in vitro gastrointestinal digestion compared to only serum incubation. This corroborates earlier studies that showed that in vitro digestion increases the uptake of 50–100 nm polystyrene particles into various blood cells, including THP-1 monocytes; however, larger particles or particles with surface modifications were not considered in that study [58]. The in vitro digestion-associated increased cellular uptake was most apparent for the PS50, PS100 and PS200 MNPs, while for the PS500 MNPs, this treatment-related difference was only significant for the lowest concentration used. The cellular uptake of both charged (PS100 (+) and PS100 (−)) MNPs was unaltered upon in vitro gastrointestinal digestion compared to serum incubation alone. For PS100 (+) confocal images suggest a preference for membrane association, while upon in vitro digestion more MNPs are seemingly internalized in the THP-1 derived macrophages. The increased propensity for membrane association of aminated positively charged MNPs can be ascribed to their higher affinity for the negatively charged cell membrane [59]. We hypothesize that the size- and charge-dependent effects of in vitro digestion found here can largely be explained by differences in the protein corona.

To verify whether the protein corona might be linked to alterations in uptake, the constituent proteins were first assessed at the global level. We first observed that the digestion-associated protein corona was sparser (i.e., fewer and more faint bands on the SDS page), more homogenous in protein composition and of lower molecular weight compared to the serum-coated corona. PS500 and PS1000 had a sparser protein corona compared to the PS50-PS200 MNPs upon incubation in serum, while for digested+serum-coated MNPs, there was no clear relation between protein abundance in the corona and size. The charge of the MNPs had a strong effect on the protein corona composition, where an increased presence of proteins in both the serum-coated, digested and digested+serum-coated PS100 (+) coronas was found, confirming previous studies reporting that increased surface charge leads to increased protein binding [60] and that surface charge is a major determinant of protein corona composition [61]. The impact of size on protein corona composition in the 100–1 µm range is poorly understood, but particle size was reported to affect both the total amount and composition of serum proteins of 50–100 nm PS particles [61]. The low variety in protein sizes in digestion coronas compared to the serum corona has previously been reported for 50 nm neutral, carboxylated and aminated polystyrene nanoplastics [24].

The reduced presence of serum proteins in the corona of digested+serum-coated MNPs indicates that the prior presence of digestion proteins impaired subsequent incorporation of serum proteins into the corona of all tested MNPs. Such a shielding effect of a preexisting protein corona has previously been applied to reduce blood clearance of targeted mesoporous silica by phagocytes [62]. The presence of a protein corona is known to reduce nonspecific interactions with the cellular membrane, which in turn reduces nonreceptor-mediated uptake [63], and studies have reported an inverse correlation between the amount of MNP-bound protein and cellular uptake [63, 64]. The reduced amount of protein bound to the digested+serum-coated PS MNPs may therefore contribute to their higher internalization compared to serum-coated PS MNPs. The larger MNPs showed a less pronounced difference in protein abundance in their corona between serum-coated and digested+serum-coated conditions, which may explain the similar cellular uptake between the different treatments of PS500 and PS1000 MNPs. Previously it has been shown that particle surface curvature can induce protein denaturation which stimulates uptake through misfolded protein receptor mediated endocytosis [21, 65, 66]. The surface curvature is inversely proportional to the squared radius of a sphere and thus larger protein denaturation is expected for small MNPs compared to large MNPs. Additionally it was shown that increased misfolding of coronated proteins was related to increased cytotoxicity in isolated blood cells, highlighting the importance of this effect [67]. Future studies assessing protein denaturation on in vitro digested MNPs and whether this causes increased recognition by misfolded protein receptors may help elucidate this effect.

To investigate whether specific proteins may drive the effect of in vitro digestion on cellular uptake, the protein corona composition was characterized in detail using LC‒MS–MS-based proteomics. The PS100, PS1000, PS100 (+) and PS100 (−) MNP subset was chosen to elucidate the influence of size and charge on the treatment-related protein corona composition. We identified 280 proteins, of which 173 proteins were found in the digested+serum-coated MNP corona, and 219 proteins were present in the serum-coated MNP corona. Based on the Pearson correlation between the protein abundance of different samples, PCA and hierarchical clustering of global protein abundance, PS100 MNPs showed the largest difference between only serum-coated and digested+serum-coated conditions. Corona on the PS100 (+) MNPs were most different in protein composition compared to all other tested MNPs, confirming the SDS‒PAGE results.

We performed differential expression analysis to identify proteins that were significantly differentially abundant between serum-coated and digested+serum-coated PS MNPs. PS100, PS100 (+), PS100 (−) and PS1000 MNPs showed 166, 174, 134 and 163 differentially abundant proteins, respectively. The majority of the differentially abundant proteins showed a higher abundance in the serum-coated MNP corona than in the digested+serum-coated MNP corona. Using a Fischers exact test, we found that the serum-coated PS100 particles were functionally enriched for proteins involved in the coagulation and complement cascade, while no other particle had a functional enrichment in the differentially abundant proteins. The coagulation and complement cascade has been intensively studied in the context of nanomedicine, and the presence of these proteins was found to increase particle phagocytosis of liposomes and PMMA nanoplastics [68, 69]. The complement proteins found on serum-coated PS100 included proteins with a known role in phagocytosis or that have previously been related to nanoparticle uptake, such as plasminogen [70, 71], complement factor C3 [69], complement factor H [68], coagulation factor XIII A [72], prothrombin [73], alpha-1-antitrypsin [73] and von Willebrand factor 74.

Next, we investigated proteins that were only differentially abundant on one particle, focusing on proteins with a known role in particle uptake. In total, we found 20, 51, 4 and 10 proteins that were only differentially present for PS100, PS100 (+), PS100 (−) and PS1000, respectively. Digested+serum-coated PS100 MNPs contained an increased amount of apolipoproteins A-I, B and D, while the serum-coated PS100 had an increased amount of the aforementioned complement proteins as well as ITIH-1 and 2, clusterin and collagen type V alpha chain. For PS100 (+), we found an increased abundance of ITIH3, ITIH4, Serpin-A3-8 and Pantetheinase in serum-coated conditions and proaposin in digested+serum-coated conditions. PS100 (−) had no unique proteins that have a known role in phagocytosis, and PS1000 had an increased abundance of mimecan and coagulation factor V in serum-coated conditions. Among the identified proteins, apolipoproteins and complement proteins have most firmly been implicated in nanoparticle uptake. Apolipoproteins have previously been shown to be involved in nanoparticle uptake [75, 76] by neutrophils and macrophages (resulting in blood clearance of MNPs) and were shown to correlate well with nanoparticle uptake in numerous studies [55, 56]. The specific differential presence of these proteins on digested+serum-coated PS100 was unexpected, as apolipoproteins originate from the serum component. The increased abundance of ITIH proteins on serum-coated PS100 and PS100 (+) is also of interest, as ITIH hyaluronan transporter proteins have previously been postulated to facilitate the binding of liposomes to the cell surface [56, 77] and were shown to correlate well with PS MNP uptake in the current study.

The proteomics dataset in our study uniquely allowed a calculation of Pearson correlation coefficients between identified corona proteins and MNP uptake by THP-1-derived macrophages to determine whether the identified proteins are related to particle uptake patterns. For 40 proteins, we found correlation coefficients > 0.5 with uptake, of which 30 were differentially abundant on at least one particle, with 14 being differentially abundant on exactly 1 particle. The most positively correlated proteins included apolipoprotein A-II, C-II and E and hemoglobin subunits a and b, while the corona proteins that inversely correlated with uptake were fetuin-b, vitronectin, neural cell adhesion molecule 1 and collagen type V alpha chain. Additionally, apolipoprotein-A1 and B, complement and coagulation factors C8, C3, V, XIII-a, fibrinogen, von Willebrand factor and prothrombin were correlated with uptake. Overall, the Pearson correlation, differential expression analysis and Fischer’s exact test support the notion that uptake differences observed between serum-coated particles and digested+serum-coated particles are driven by members of the coagulation and complement cascade and apolipoproteins such as opsonins. The proteins identified here show good concordance with previous studies and known roles in endocytotic processes [55, 56, 68–73]. Thus, they represent a likely cause for the difference in particle uptake between digested+serum-coated and serum-coated PS MNPs. The proteins identified in the protein coronas of our studied MNPs that had a well-established relationship with phagocytosis were differentially abundant on PS100 but also on the larger PS1000 MNPs. We, however, did not observe striking treatment-related PS1000 MNP uptake differences. Potentially, the relative importance of the protein corona for PS1000 for uptake is less pronounced than that for PS100, which was also indicated by the reduced number of proteins in the larger PS MNPs. This is supported by previous studies that reported that increasing particle size reduces the effect of surface functionalization on particle clearance by macrophages [78, 79].

The current study shows that the in vitro gastrointestinal digestion-associated protein corona increases the uptake of PS MNPs in THP-1-derived macrophages in an MNP size- and charge-dependent manner. It was shown that in vitro intestinal digestion alters the binding of serum proteins on these MNPs, but the mechanism behind this is still unclear. It might be possible that the presence of digestion proteins in the corona causes steric hindrance for serum proteins or competition for binding of serum proteins in terms of affinity. Alternatively, digestion-related proteases present on the surface of MNPs are partially shielded from serum-borne protease inhibitors such as serpins and might actively degrade serum proteins bound to the surface. Based on the retention of digestive proteins on MNPs, it can be predicted that proteins from food components might also be retained on MNPs; however, this has not been studied thus far. The retention of food-bound components on MNPs may hold relevance to food immunity and warrants further studies. In addition, humans are exposed through their diet to MNPs with different polymer types, such as PET, PE and PP, as well as MNPs weathered by the environment. Little is known on the physicochemical characteristics of environmental MNPs, however larger plastic fragments are known to undergo photodegradation, biodegradation and mechanical abrasion ultimately resulting in MNPs [80–82]. Artificial weathering of MNPs resulted in an increased hydrophilicity of such MNPs compared to pristine MNP, which may alter the protein corona formation and thus the cellular uptake of environmental MNPs [83, 84]. Furthermore environmental components comprising the so-called eco corona may also increase cellular uptake [85]. Further studies investigating the role of MNP polymer type, environmental weathering and food components present during (in vitro) gastrointestinal digestion on nanoparticle uptake can help elucidate these questions [86, 87].

Based on the LC‒MS–MS-based proteomics results, we postulate that altered binding of complement and coagulation cascade proteins to nanoplastics caused the increased uptake of nanoplastics after in vitro digestion. The alteration in complement and coagulation components was less pronounced in charged PS MNPs or in MNPs of 1000 nm, resulting in a minimal effect of digestion on their uptake. The increased uptake of digested MNPs may lead to increased mitochondrial membrane potential, of macrophages as an increased internal dose (of 40–90 nm 0.7–0.9 µm and polystyrene MNPs) has been associated with perturbed mitochondrial potential, ROS production and alterations in surface marker expression of J774A.1 mouse macrophages [88], although other studies revealed no obvious increase in toxicity after THP-1 monocyte exposure to in vitro digested 50–100 nm PS MNPs [58].

## Conclusions

We have shown that in vitro digestion significantly alters the uptake of 50 and 100 nm uncharged PS MNPs by THP-1-derived macrophages but not charged 100 nm MNPs or MNPs larger than 500 nm. We found that the presence of digestion proteins in the protein corona alters the subsequent binding of serum components to MNPs. Forty proteins were correlated with uptake. The proteins identified here have known roles in phagocytosis, and apolipoproteins such as A-1, A-II, and C-II and complement and coagulation cascade proteins such as von Willebrand factor, complement C3 and fibrinogen may cause differences in uptake. This is the first study that quantitatively measured the effect of in vitro digestion on micro- and nanoplastic uptake in THP-1-derived macrophages, linking this to alterations to the protein corona. Our results indicate that digestion should be taken into account when estimating cellular uptake, and using serum-coated MNPs can lead to a significant underestimation of MNP internalization by cells.

### Supplementary Information

**Additional file 1. **Supplementary figures.

**Additional file 2. **Raw proteomics data and differential expression analysis.

## Data Availability

The datasets used and/or analyzed during the current study are available from the corresponding author upon reasonable request.

## Abbreviations

MNPs: Micro- and nanoplastics
PS: Polystyrene
SSF: Simulated salivary fluid
SGF: Simulated gastric fluid
SIF: Simulated intestinal fluid
CCM: Complete cell culture medium
DLS: Dynamic light scattering
ISDD: In vitro sedimentation, diffusion and dosimetry
PMA: Phorbol 12-myristate 13-acetate
WST-1: Water-soluble tetrazolium dye 1
FITC: Fluorescein isothiocyanate
RLU: Relative light units
SDS‒PAGE: Sodium dodecyl sulfate–polyacrylamide gel electrophoresis
LC‒MS–MS: Liquid chromatography tandem mass spectrometry
HCD: Higher-energy C-trap dissociation
LFQ: Label-free quantification
FDR: False discovery rate
PDI: Polydispersity index
