# Supplementary material for: The in vitro gastrointestinal digestion-associated protein corona of polystyrene nano- and microplastics increases their uptake by human THP-1-derived macrophages
Source: Part Fibre Toxicol. 2024 Feb 4;21:4. doi: 10.1186/s12989-024-00563-z (PMC10838446; doi:10.1186/s12989-024-00563-z)
Supplement: Supplementary file 1 — Additional file 1. Supplementary figures. [file 12989_2024_563_MOESM1_ESM.docx]

***Supplementary information: In vitro* gastrointestinal digestion associated protein corona of nano- and microplastics affects their uptake by human THP-1 derived macrophages**

Hugo Brouwer^a^, Laura de Haan^a^, Mojtaba Porbahaie^c^, Sjef Boeren^b^, Ivonne Rietjens^a^, Hans Bouwmeester^a^

a. Division of Toxicology, Wageningen University, The Netherlands.

b. Laboratory of biochemistry, Wageningen University,

c. Laboratory of cell biology and immunology, Wageningen University,

*to be submitted to:* *Journal of hazardous materials*

*Corresponding author:

Hugo Brouwer, Division of Toxicology, Wageningen University

Stippeneng 4, 6708 WE Wageningen, The Netherlands.

E-mail: [Hugo.brouwer@wur.nl](mailto:Hugo.brouwer@wur.nl)

**Supplementary information:**

**
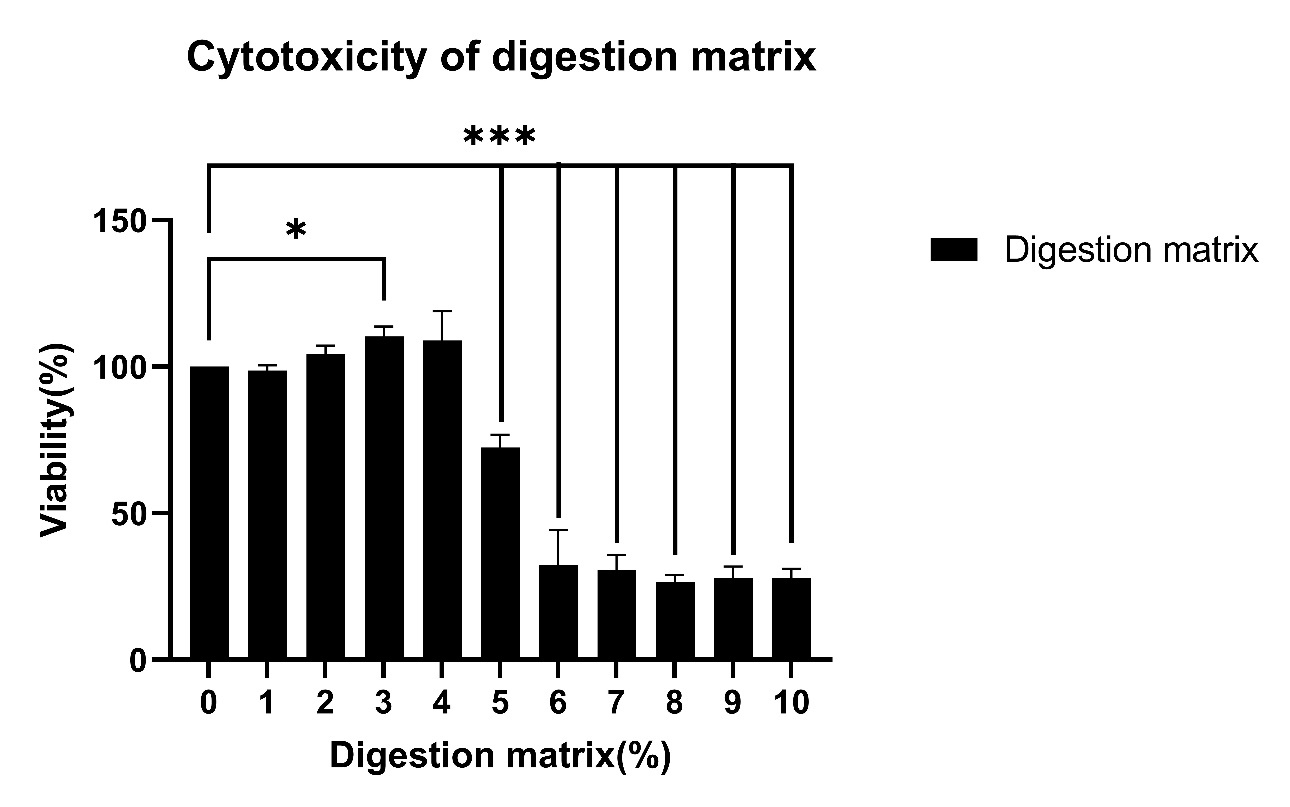
**

**Fig. S1: digestion matrix toxicity.**

*The y-axis shows relative viability of THP-1 derived macrophages after exposure to 1-10 % of digestion matrix diluted in serum-containing medium as measured using WST-1. Viability is expressed in the relative absorption at 440nm compared to the medium control. The error bars show the standard deviation of the measurement. A two tailed ANOVA using Dunnett’s post-hoc correction was performed to assess significant differences from the medium control, n=3.* *Significance is indicated: * ( P<0.05) ^**^(P<0.01), ***(P<0.001).*

**
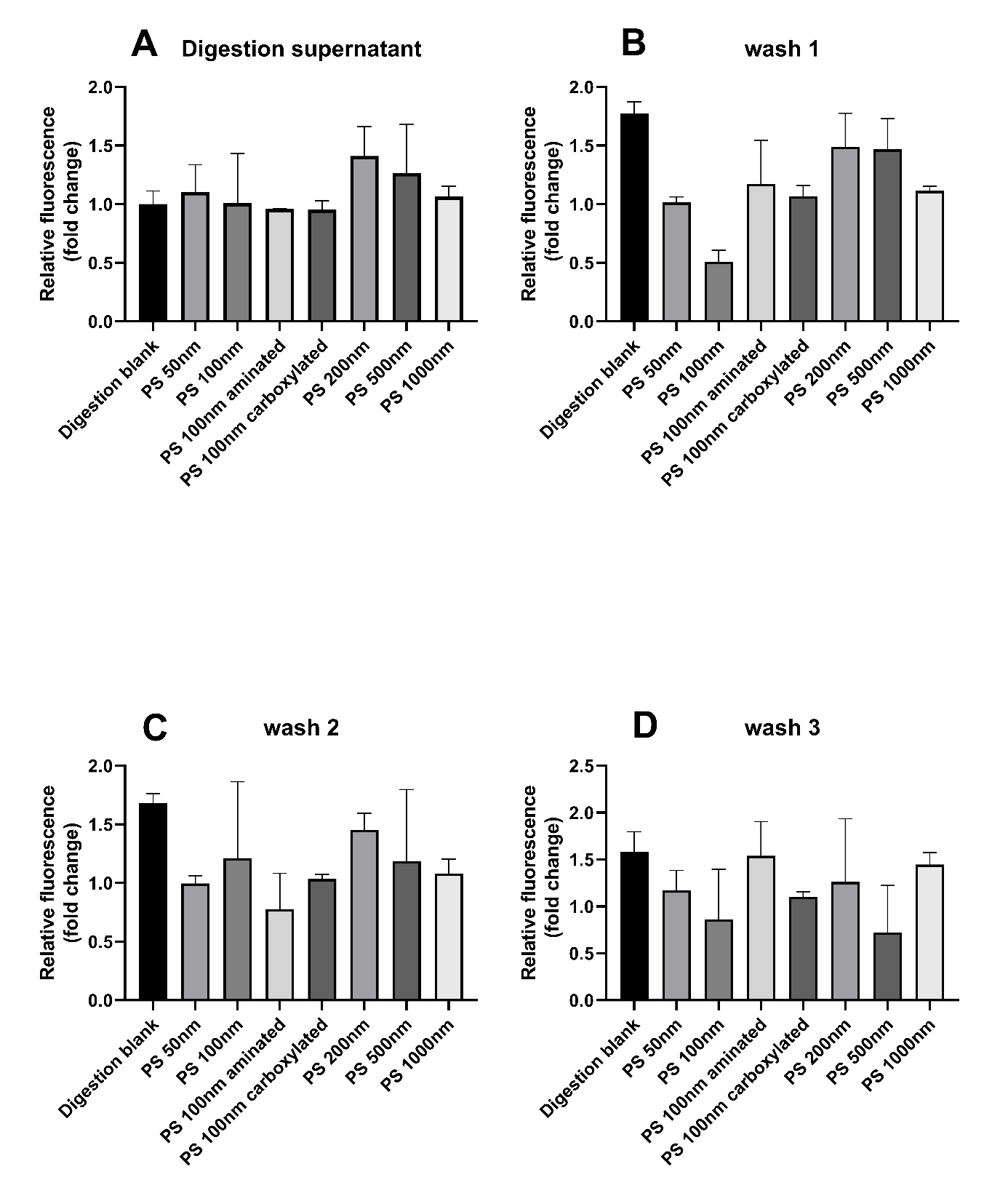
**

**
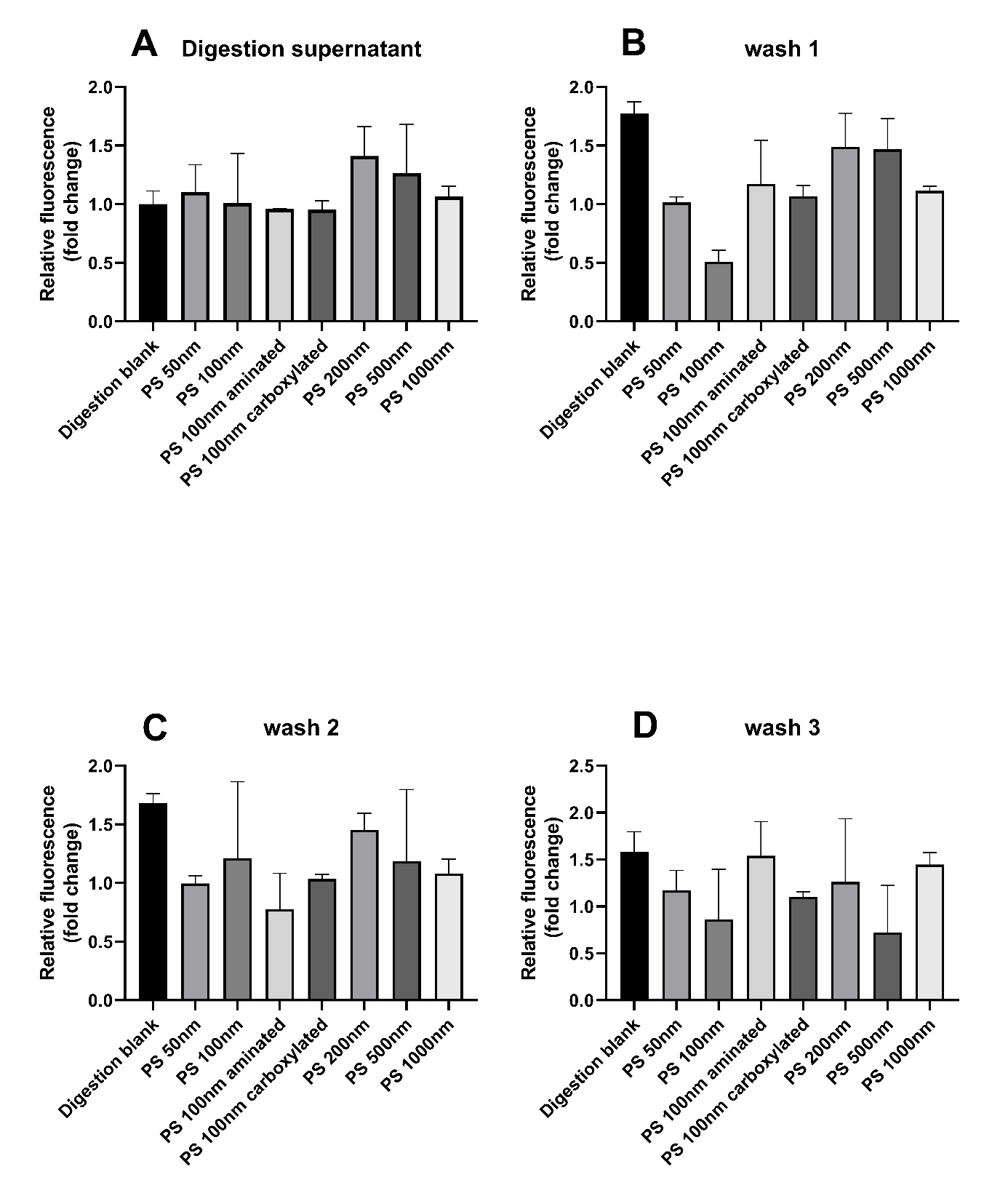
 Fig. S2: Fluorophore leaching after digestion and subsequent washes**

*The y-axis shows the relative fluorescence measured after 30 minutes of centrifugation at 30,000 RCF in (A) the supernatant of the digestion, (B) the supernatant after the 1^st^ PBS wash, (C) the supernatant after the 2^nd^ PBS wash and (D) the supernatant after the 3^rd^ PBS wash. The excitation was set at 485nm while the emission was measured at 525 nm using a Promega spectramax iD3 luminometer. the Y-axis shows the fold change of each sample compared to the digestion blank in graph A.*


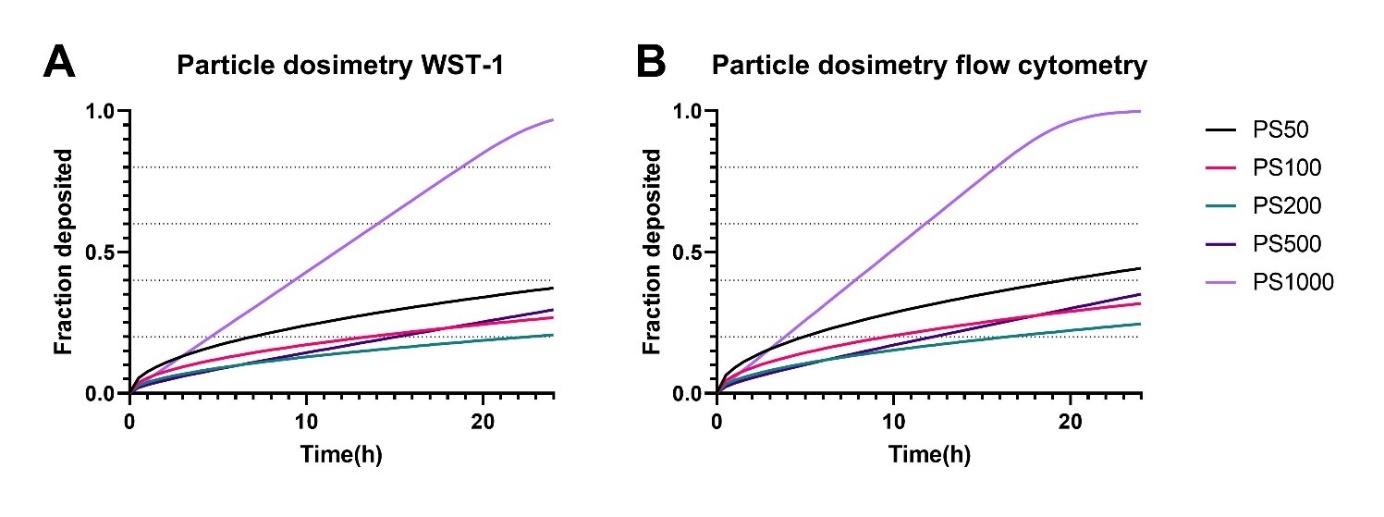


**Fig. S3: Particle dosimetry for cell exposure to micro/nanoplastics**

*The predicted particle sedimentation during the WST-1 assay and Flow-cytometry based particle adhesion tests. The Y-axis shows the fraction of total particles that are deposited and the X-axis shows the time in hours.*


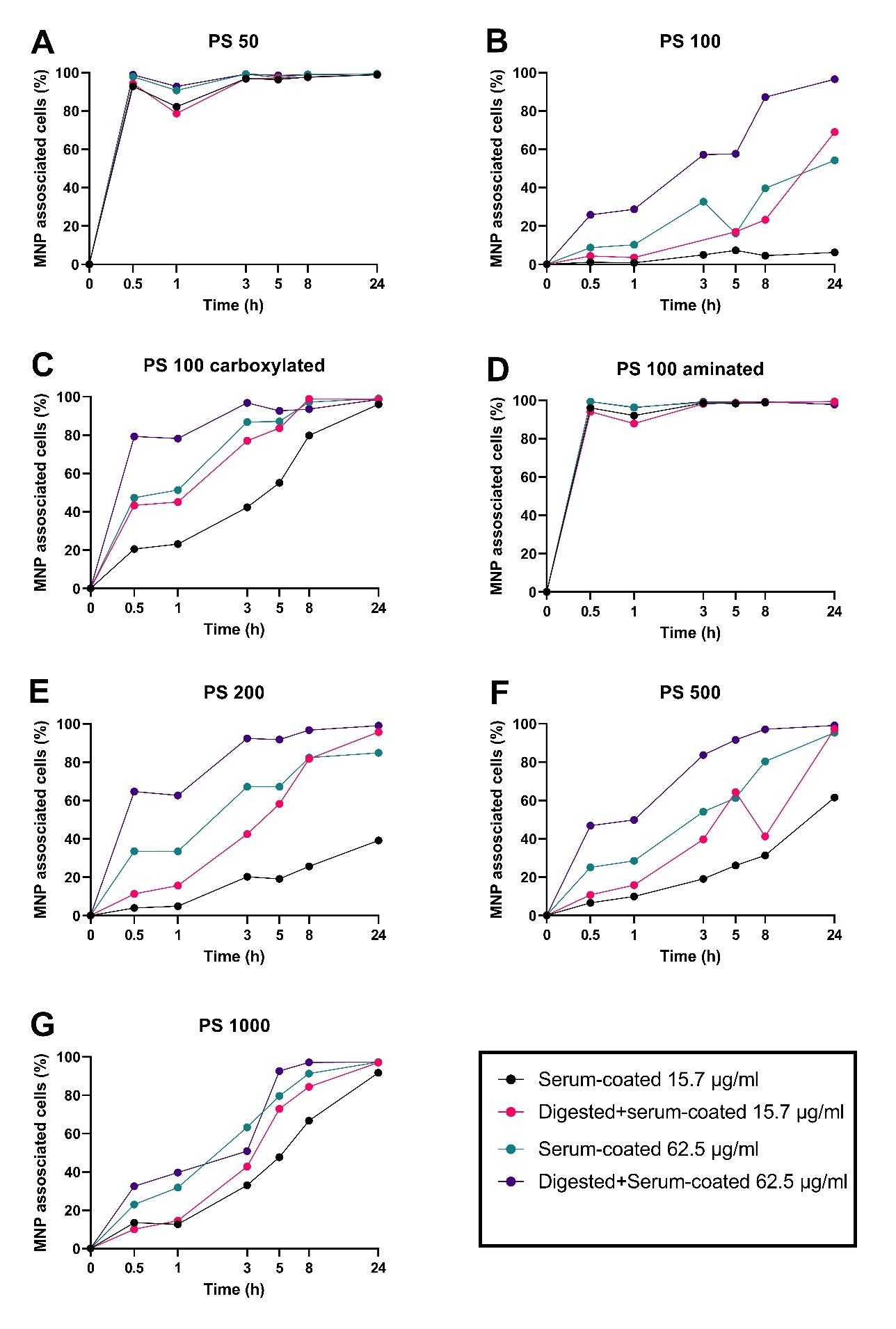


**Fig. S4:** *Percentage of THP-1 derived macrophages associated with MNPs in time.* *MNP cell association after 0.5,1,3,5,8 or 24 h exposure to digested+serum-coated or serum-coated MNPs at a concentration of 15.7 or 62.5 µg/ml. The graphs indicate association of (A) PS 50, (B) PS 100, (C) aminated PS 100, (D) carboxylated PS 100, (E) PS 200, (F) PS 500, (G) PS 1000. The y-axis indicate the percentage of cells associated with at least 1 fluorescent particle, defined as having a fluorescence intensity higher than 99% of the unexposed cells. n=1*


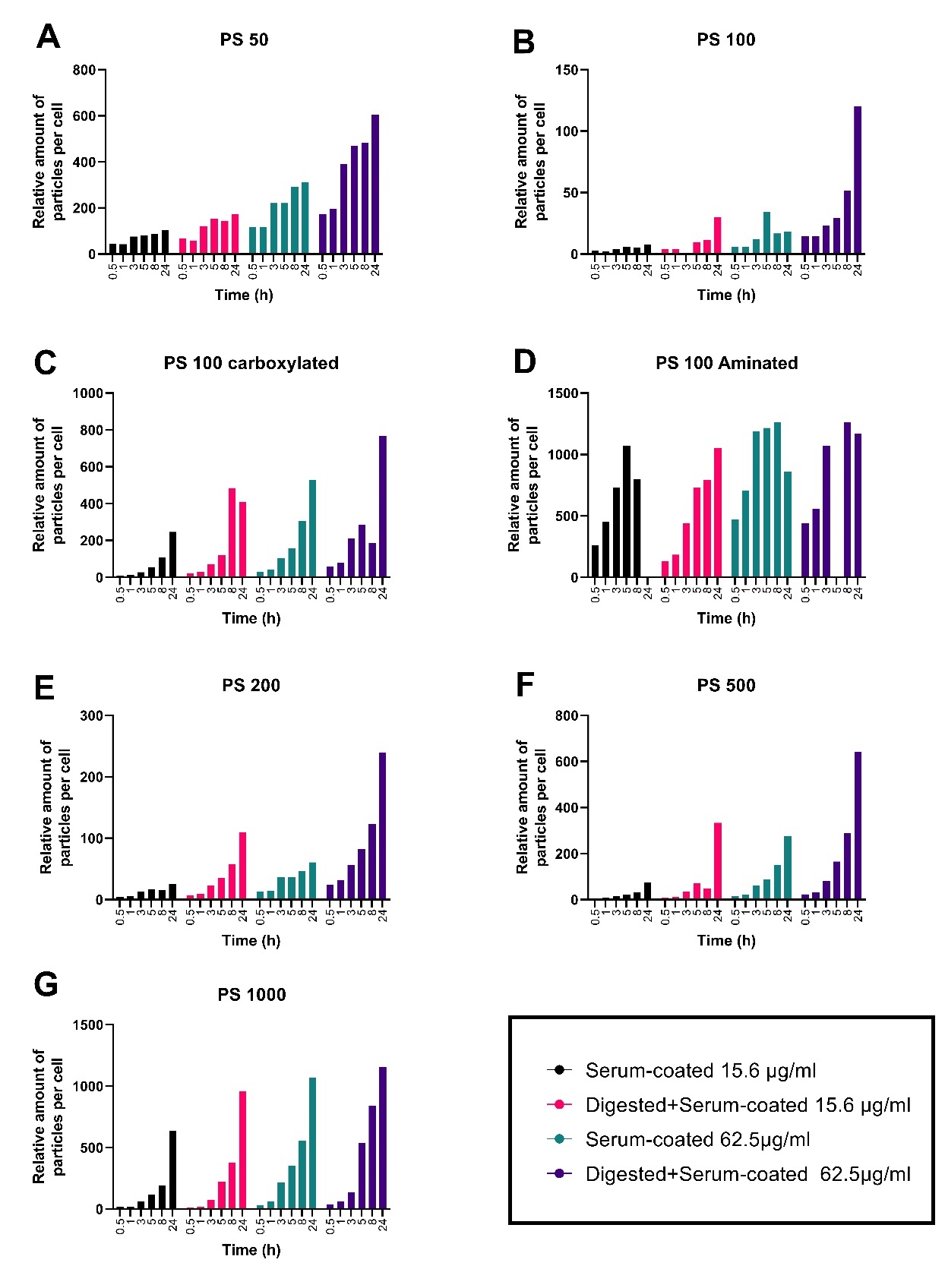


**Fig. S5:** *Amount of MNPs associated with THP-1 derived macrophages in time. MNP cell association after 0.5,1,3,5,8 or 24 hour exposure to digested or serum-coated MNPs. The bar graphs show the average relative fluorescence originating from MNPs per cell after 0.5,1,3,5,8 or 24 hour exposure to digested or serum-coated MNPs.* *The graphs indicate association of (A) PS 50, (B) PS 100, (C) aminated PS 100, (D) carboxylated PS 100, (E) PS 200, (F) PS 500, (G) PS 1000. The relative fluorescent intensity is the fold change in the 525:20 channel compared to the unexposed cells. The cells were exposed to either 15.6 µg/ml or 62.5 µg/ml of MNPs. The legend on the left indicates the concentration and particle treatment. n=1*


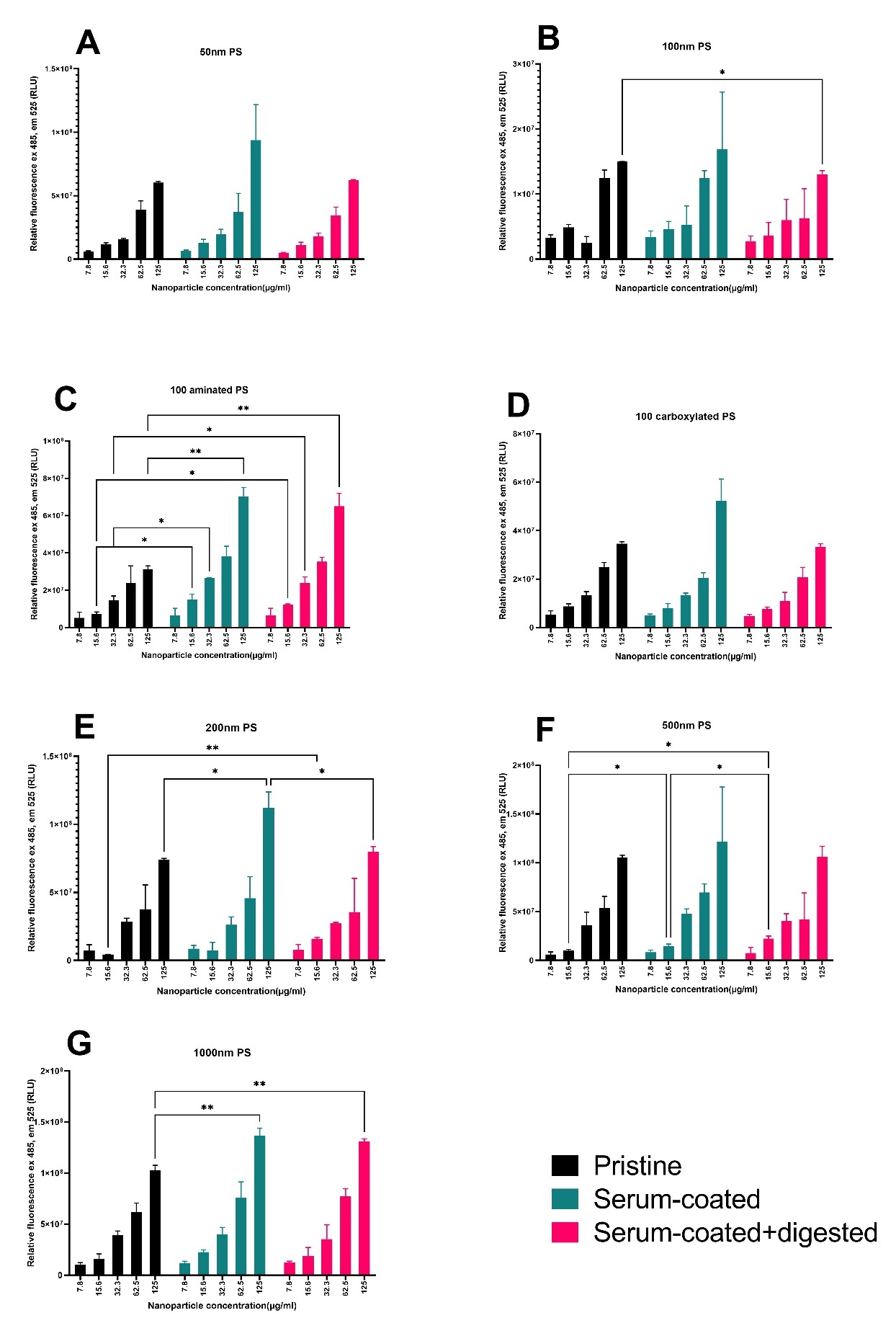


**Fig. S6: Relative luminescence of pristine, serum coated and digested particles**

*This figure shows the relative fluorescence of pristine, serum-coated and serum-coated+digested particles. The excitation was at 485nm while the emission was measured at 525 nm. The figures show the fluorescence of (A) 50nm PS, (B) 100nm PS, (C) 100nm aminated PS, (D) 100nm carboxylated PS, (E) 200nm PS, (F) 500nm PS, (H) 1000nm PS. Fluorescence was measured using a spectramax iD3 luminometer*

Supplementary table S1:Proteins uniquely enriched in any of the conditions

| Unique to | Protein name | Abbreviation | Literature |
| --- | --- | --- | --- |
| Serum-coated PS100 | Coactosin-like protein | Q2HJ57 | (1) |
| Serum-coated PS100 | Collagen type V alpha 1 chain | G3MZI7 | (2) |
| Serum-coated PS100 | von Willebrand factor | P80012 | (3) |
| Serum-coated PS100 (+) | Leucine rich alpha-2-glycoprotein 1 | F6RMV5 | - |
| Serum-coated PS100 (+) | Serpin domain-containing protein | A0A0A0MPA0 | - |
| Serum-coated PS100 (+) | 60S acidic ribosomal protein P0 | A0A287AY54 | - |
| Serum-coated PS100 (+) | Inositol-3-phosphate synthase 1 | A0A287BDF8 | - |
| Serum-coated PS100 (+) | SERPINA11 protein | A5PK77 | - |
| Serum-coated PS100 (+) | SHBG protein | A5PKC2 | - |
| Serum-coated PS100 (+) | Serpin A3-8 | A6QPQ2 | (3) |
| Serum-coated PS100 (+) | Gamma-glutamyl hydrolase | A7YWG4 | - |
| Serum-coated PS100 (+) | Corticosteroid-binding globulin | E1BF81 | (3) |
| Serum-coated PS100 (+) | Peptidoglycan recognition protein 2 | E1BH94 | - |
| Serum-coated PS100 (+) | Alpha-amylase, 3.2.1.1 | F1MJQ3 | (1) |
| Serum-coated PS100 (+) | Mannose receptor C type 2 | F1MPD1 | - |
| Serum-coated PS100 (+) | CD109 molecule | F1MPE1 | - |
| Serum-coated PS100 (+) | Actinin alpha 3 | F1RU49 | - |
| Serum-coated PS100 (+) | Ig-like domain-containing protein | G5E5T5 | (4)  (5) |
| Serum-coated PS100 (+) | MBL associated serine protease 2 | I2E4T6 | - |
| Serum-coated PS100 (+) | Osteomodulin | O77742 | (3) |
| Serum-coated PS100 (+) | Fibromodulin | P13605 | (1) |
| Serum-coated PS100 (+) | Insulin-like growth factor binding protein 2 | Q09TE3 | (6) |
| Serum-coated PS100 (+) | Pantetheinase | Q58CQ9 | (3, 6) |
| Serum-coated PS100 (+) | Acidic mammalian chitinase | Q95M17 | (3, 6, 7) |
| Serum-coated PS1000 | pyruvate kinase M1/2 | F1SHM0 | - |
| Serum-coated PS1000 | Protein-lysine 6-oxidase | P33072 | (1) |
| Serum-coated PS1000 | Protein HP-20 homolog | Q2KIT0 | (3) |
| Serum-coated PS1000 | Parkinson disease protein 7 homolog | Q5E946 | - |
| Digested+serum-coated PS100 | Ig-like domain-containing protein | A0A3Q1M1Z4 | - |
| Digested+serum-coated PS100 (+) | Peptidyl-prolyl cis-trans isomerase | A0A286ZKG9 | - |
| Digested+serum-coated PS100 (+) | 60S ribosomal protein L14 | A0A286ZW72 | - |
| Digested+serum-coated PS100 (+) | 40S ribosomal protein S15a | A0A287A014 | - |
| Digested+serum-coated PS100 (+) | Nucleoside diphosphate kinase | A0A5G2Q9S0 | - |
| Digested+serum-coated PS100 (+) | Ribosomal protein S3 | A0A5G2QSB4 | - |
| Digested+serum-coated PS100 (+) | KDEL endoplasmic reticulum protein retention receptor 2 | A0A5G2R5F8 | - |
| Digested+serum-coated PS100 (+) | Hydroxysteroid 17-beta dehydrogenase 13 | F1RW28 | - |
| Digested+serum-coated PS100 (+) | Staphylococcal nuclease domain-containing protein | F1SML4 | - |
| Digested+serum-coated PS100 (+) | Phosphate carrier protein, mitochondrial | F1SQT3 | - |
| Digested+serum-coated PS100 (+) | Endoplasmic reticulum protein 27 | F1SQW6 | - |
| Digested+serum-coated PS100 (+) | 40S ribosomal protein S11 | F2Z4Y8 | - |
| Digested+serum-coated PS100 (+) | Peroxiredoxin 4 | K7GLN4 | - |
| Digested+serum-coated PS100 (+) | 60S ribosomal protein L8 | K7GNY4 | - |
| Digested+serum-coated PS100 (+) | Ig lambda chain C region | P01846 | (8) |
| Digested+serum-coated PS100 (+) | Prosaposin | P26779 | (9) |
| Digested+serum-coated PS100 (+) | Cytosol aminopeptidase | P28839 | - |
| Digested+serum-coated PS100 (+) | ATP synthase subunit alpha | P80021 | - |
| Digested+serum-coated PS100 (+) | Dolichyl-diphosphooligosaccharide--protein glycosyltransferase | Q29036 | - |
| Digested+serum-coated PS100 (+) | Large ribosomal subunit protein eL8 | Q29375 | - |
| Digested+serum-coated PS100 (-) | Palmitoyl-protein thioesterase 1 | A0A287AYH9 | - |
| Digested+serum-coated PS100 (-) | Ig-like domain-containing protein | F1MZ96 | - |
| Serum-coated PS1000 | pyruvate kinase M1/2 | F1SHM0 | - |
| Serum-coated PS1000 | Protein-lysine 6-oxidase | P33072 | (1) |
| Serum-coated PS1000 | Protein HP-20 homolog | Q2KIT0 | (3) |
| Serum-coated PS1000 | Parkinson disease protein 7 homolog | Q5E946 | - |
|  |  |  |  |

Proteins uniquely identified on one of the MNP protein coronas using LC-MS-MS. The Literature column indicates sources which have previously implied the protein as being assosciated with altered uptake of nanomaterials or altered phagocytosis.


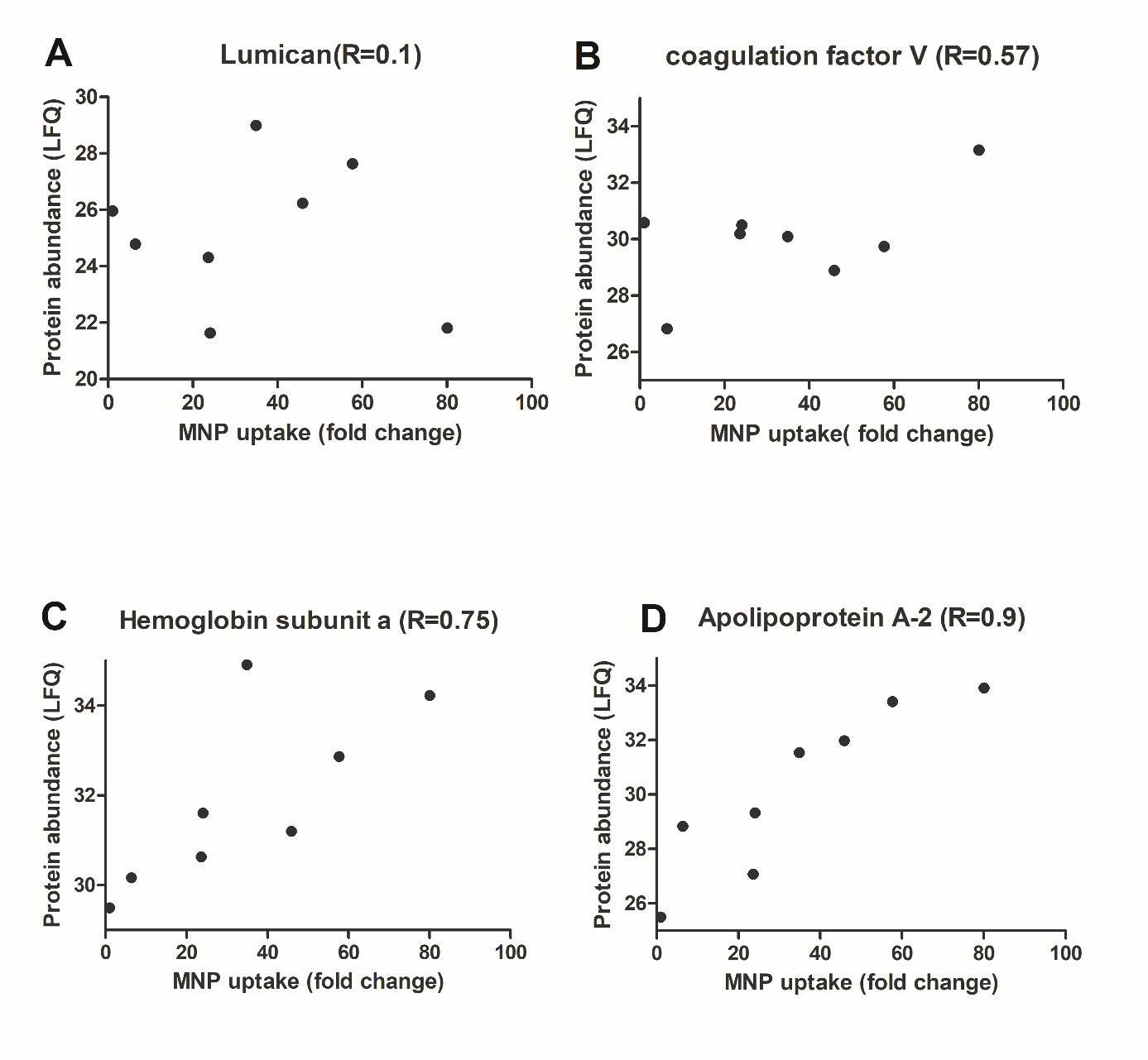


**Fig. S7: Overview of correlation patterns of poorly, moderately and well correlated proteins**

*This figure shows the correlation of the protein abundance in LFQ with the fold change in MNP uptake for (A) Lumican, (B) Coagulation factor V, (C) Hemoglobin subunit a and (D) Apolipoprotein A-2. The R-value at the title of each graph indicates how high the pearson correlation between protein abundance and uptake was with R=1 being perfectly correlated and R=0 having no correlation.*


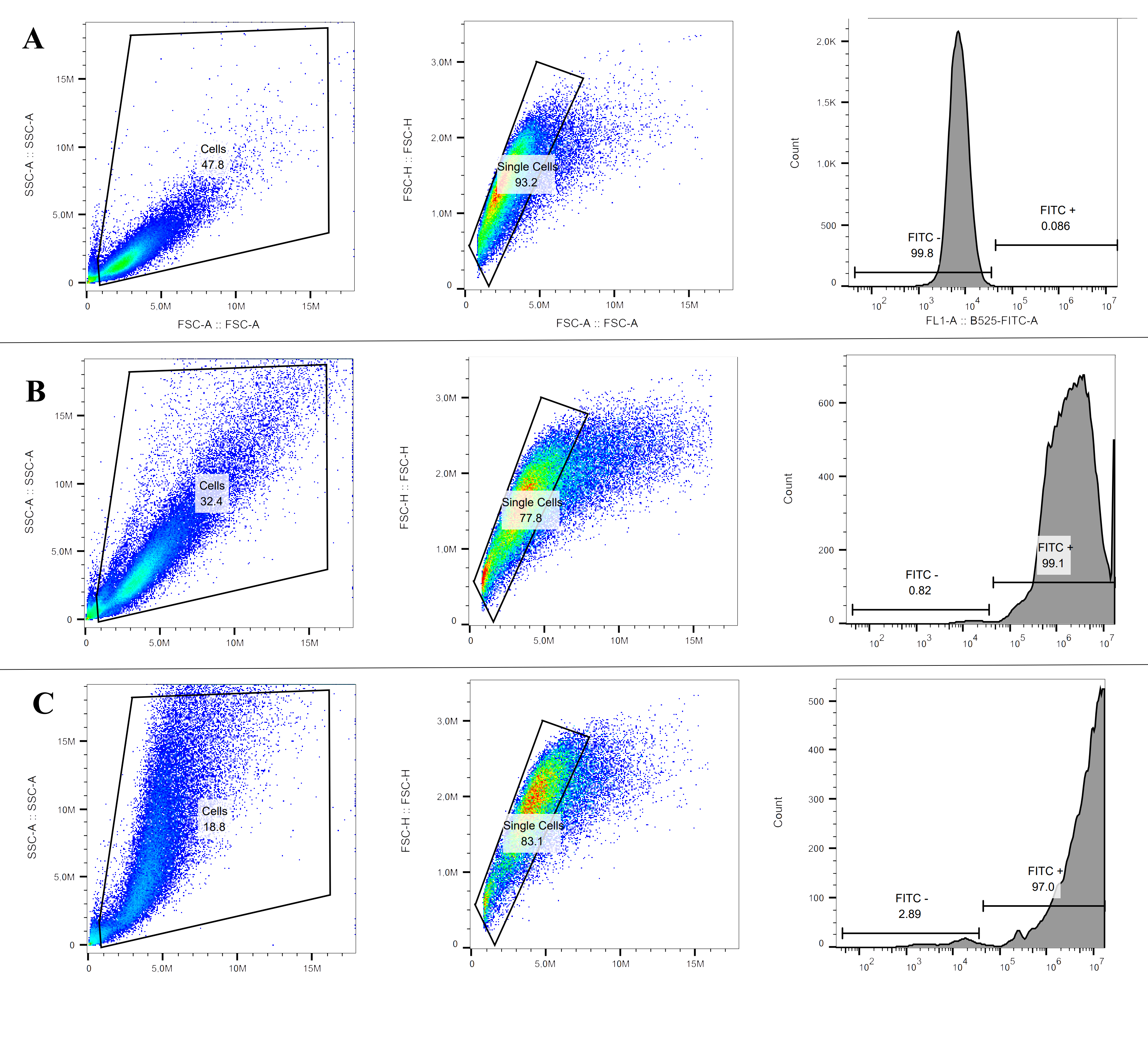


**Fig. S8: Flow cytometry sorting strategy and alterations in forward side scatter after treatment**

*This figure shows the sorting strategy to determine the mean fluorescence intensity of cells exposed to A) cell culture medium, B) cell culture medium and 31.3 µg/ml of digested+serum coated PS50 and C) 31.3 µg/ml digested+serum-coated PS1000. The first column of figures show the gating to separate cells from debris, the y-axis shows the side-scatter area and the X-axis shows the forward scatter area. The second column of figures shows the isolation of singlet cells, the y-axis shows the forward scatter height and the X-axis shows the forward scatter area. The third column of figures shows a histogram of the particle fluorescence in single cells, the y-axis shows the count and the X-axis shows the area of the fluorescence at 525/20 nm.*

1. Miclăuş T, Beer C, Chevallier J, Scavenius C, Bochenkov VE, Enghild JJ, et al. Dynamic protein coronas revealed as a modulator of silver nanoparticle sulphidation in vitro. Nature Communications. 2016;7(1):11770.

2. Jiang P, Zhu Y, Kang K, Luo B, He J, Wu Y. Protein corona of magnetic PEI/siRNA complex under the influence of a magnetic field improves transfection efficiency via complement and coagulation cascades. Journal of Materials Chemistry B. 2019;7(26):4207-16.

3. Oh JY, Kim HS, Palanikumar L, Go EM, Jana B, Park SA, et al. Cloaking nanoparticles with protein corona shield for targeted drug delivery. Nature Communications. 2018;9(1):4548.

4. Shannahan JH, Lai X, Ke PC, Podila R, Brown JM, Witzmann FA. Silver nanoparticle protein corona composition in cell culture media. Plos One. 2013;8(9):e74001.

5. Arezki Y, Delalande F, Schaeffer-Reiss C, Cianférani S, Rapp M, Lebeau L, et al. Surface charge influences protein corona, cell uptake and biological effects of carbon dots. Nanoscale. 2022;14(39):14695-710.

6. Galmarini S, Hanusch U, Giraud M, Cayla N, Chiappe D, von Moos N, et al. Correction to Beyond Unpredictability: The Importance of Reproducibility in Understanding the Protein Corona of Nanoparticles. Bioconjugate Chemistry. 2019;30(6):1832-.

7. Pisani C, Gaillard J-C, Odorico M, Nyalosaso JL, Charnay C, Guari Y, et al. The timeline of corona formation around silica nanocarriers highlights the role of the protein interactome. Nanoscale. 2017;9(5):1840-51.

8. Lundqvist M, Stigler J, Elia G, Lynch I, Cedervall T, Dawson KA. Nanoparticle size and surface properties determine the protein corona with possible implications for biological impacts. P Natl Acad Sci USA. 2008;105(38):14265-70.

9. Qin M, Zhang J, Li M, Yang D, Liu D, Song S, et al. Proteomic analysis of intracellular protein corona of nanoparticles elucidates nano-trafficking network and nano-bio interactions. Theranostics. 2020;10(3):1213-29.
